# Identification of hub genes and potential molecular mechanisms related to drug sensitivity in acute myeloid leukemia based on machine learning

**DOI:** 10.3389/fphar.2024.1359832

**Published:** 2024-04-08

**Authors:** Boyu Zhang, Haiyan Liu, Fengxia Wu, Yuhong Ding, Jiarun Wu, Lu Lu, Akhilesh K. Bajpai, Mengmeng Sang, Xinfeng Wang

**Affiliations:** ^1^ Department of Hematology, Affiliated Hospital of Nantong University, Medical School of Nantong University, Nantong, Jiangsu, China; ^2^ Department of Genetics, Genomics, and Informatics, University of Tennessee Health Science Center, Memphis, TN, United States

**Keywords:** LAML, machine learning, prognostic models, predicting drugs, molecular docking

## Abstract

**Background:** Acute myeloid leukemia (AML) is the most common form of leukemia among adults and is characterized by uncontrolled proliferation and clonal expansion of hematopoietic cells. There has been a significant improvement in the treatment of younger patients, however, prognosis in the elderly AML patients remains poor.

**Methods:** We used computational methods and machine learning (ML) techniques to identify and explore the differential high-risk genes (DHRGs) in AML. The DHRGs were explored through multiple *in silico* approaches including genomic and functional analysis, survival analysis, immune infiltration, miRNA co-expression and stemness features analyses to reveal their prognostic importance in AML. Furthermore, using different ML algorithms, prognostic models were constructed and validated using the DHRGs. At the end molecular docking studies were performed to identify potential drug candidates targeting the selected DHRGs.

**Results:** We identified a total of 80 DHRGs by comparing the differentially expressed genes derived between AML patients and normal controls and high-risk AML genes identified by Cox regression. Genetic and epigenetic alteration analyses of the DHRGs revealed a significant association of their copy number variations and methylation status with overall survival (OS) of AML patients. Out of the 137 models constructed using different ML algorithms, the combination of Ridge and plsRcox maintained the highest mean C-index and was used to build the final model. When AML patients were classified into low- and high-risk groups based on DHRGs, the low-risk group had significantly longer OS in the AML training and validation cohorts. Furthermore, immune infiltration, miRNA coexpression, stemness feature and hallmark pathway analyses revealed significant differences in the prognosis of the low- and high-risk AML groups. Drug sensitivity and molecular docking studies revealed top 5 drugs, including carboplatin and austocystin-D that may significantly affect the DHRGs in AML.

**Conclusion:** The findings from the current study identified a set of high-risk genes that may be used as prognostic and therapeutic markers for AML patients. In addition, significant use of the ML algorithms in constructing and validating the prognostic models in AML was demonstrated. Although our study used extensive bioinformatics and machine learning methods to identify the hub genes in AML, their experimental validations using knock-out/-in methods would strengthen our findings.

## Introduction

Acute myeloid leukemia (AML) is the most common leukemia among adults and accounts for nearly 80% of all cases (Yamamoto and Goodman, 2008; Shimony et al., 2023). It is a heterogenous disease and is characterized by uncontrolled proliferation and clonal expansion of hematopoietic cells resulting in ineffective erythropoiesis and bone marrow failure (Shimony et al., 2023; Papaemmanuil et al., 2016; D’Kouchkovsky and Abdul-Hay, 2016). The estimated five-year survival rate varies greatly between different age groups, ranging from ∼50% in the younger patients to less than 10% in patients of 60-years age and older (Sasaki et al., 2021; Shimony et al., 2023). In the United States, the incidence of AML is about 3–5 cases per 100,000 population, and it increases with age, with ∼12 cases in older patients per 100,000 population. Males are more predominantly affected compared to females, with a ratio of 5:3(D’Kouchkovsky and Abdul-Hay, 2016; Siegel et al., 2015). The pathophysiology of AML involves multiple factors, such as radiation, chromosomal aberrations, and existing hematopoietic disorders; however, the primary cause of the disease is recurrent genetic mutations. More than 90% of AML patients harbor somatic mutations in several genes including those associated with hematopoiesis. Some of the frequently mutated genes in AML include *DNMT3A*, *IDH1*, *IDH2*, *TET2, FLT3,* and *NPM1* (Papaemmanuil et al., 2016; Angenendt et al., 2019; Kantarjian et al., 2021). Although significant improvements in the treatment of AML have been witnessed in younger patients, prognosis in the elderly, the majorly affected group remains poor (D’Kouchkovsky and Abdul-Hay, 2016; Shah et al., 2013). Therefore, it is important to gain better insights into the molecular mechanisms associated with AML and identify candidate genes for improving therapeutic strategies and disease prognosis.

Advancement in machine learning (ML) techniques and methods is fueling drug discovery and healthcare research in a large way. ML algorithms are extensively used in today’s healthcare research for disease diagnosis, discovering potential prognostic biomarkers and drug targets in various pathophysiological conditions starting from viral infections to neurodegeneration disorders (Barman et al., 2019; Alamro et al., 2023; Taheri and Habibi, 2023; Turki and Taguchi, 2023). A few of the popular ML techniques/algorithms used in biological research include support vector machine (SVM) (Noble, 2006), artificial neural network (ANN), random forest (RF), and gradient boosting tree (GBT). Alamro et al., (Alamro et al., 2023), used ranking and feature selection methods to first shortlist the hub genes associated with Alzheimer’s disease (AD) and then employed ML and deep learning (DL) methods to differentiate between AD patients and healthy controls using the selected gene-sets. Taheri et al., Taheri and Habibi, (2023) focused on a more recent problem and used three different unsupervised learning algorithms to rank the important genes and finally identified a set of 18 key genes related to COVID-19 disease. Another study that claims to be the first of its kind developed an ML-based classification approach to discover infectious disease-associated host genes and achieved the highest accuracy for a deep neural network (DNN) model with 16 selected features (Barman et al., 2019). A study by Huang et al. (2022) used bioinformatics methods along with SVM recursive feature elimination (SVM-RFE) and RF algorithms to identify hub genes in coronary artery disease. Our group recently used non-negative matrix factorization (NMF) to show that this method significantly improves the enrichment detection of glaucoma genes over the traditional differential gene expression analysis. Further, application of NMF with the scoring method developed by us showed great promise in the identification of marker genes for glaucoma, with its potential applicability to other conditions and diseases (Huang et al., 2023).

The ML techniques in the diagnosis of hematologic malignancies were used two decades ago (Zong et al., 2006); however, limitations in computational power and unavailability of large-scale data, such studies were not pursued widely. More recently, ML techniques and methods are becoming popular in the diagnosis and prognosis of AML, fortunately, due to freely available multi-omics online data sets, such as Leukemia Gene Atlas (Hebestreit et al., 2012) and The Cancer Genome Atlas [TCGA, Weinstein et al. (2013)]. Lee et al. (2018) proposed a computational approach to identify robust molecular markers for targeted treatment of AML by integrating multi-omics data from 30 patients and *in vitro* sensitivity data corresponding to 160 chemotherapy drugs. (Warnat-Herresthal et al., 2020). combined multi-omics data including transcriptomic and genomic data to develop ML classifiers that can accurately detect AML in a near-automated and low-cost method. The integration of ML with feature selection methods and comparison of their performances showed that GBT with an accuracy of >85%, AUC >0.90, and the feature selection via the Relief algorithm had the best outcome in predicting the survival rate of AML patients (Karami et al., 2021). Analyzing the genomic data from a multicenter cohort of ∼6800 AML patients, the researchers were able to decipher a set of prognostic subgroups predictive of survival using the recent ML techniques over the traditional methods (Awada et al., 2021). A review by Eckardt et al., (Eckardt et al., 2020) discusses in detail the applications of various ML methods and algorithms in the diagnosis, prognosis, and treatment of AML. The use of ML in understanding AML is comparatively newer and provides a lot of opportunities to use this method in exploring the disease in detail.

In the current study, firstly, we identified high-risk genes in AML using various genomic and functional analysis approaches. We then developed a consensus ML-driven signature using the high-risk genes and different algorithms and selected the best prognostic model. The prognostic significance of the high-risk genes was further evaluated using survival analysis, and independent training and validation cohorts. The immune infiltration, miRNA co-expression and stemness features analysis of the high-risk genes confirmed the importance of this gene-set in AML prognosis and survival. Lastly, using the molecular docking studies, we identified potential drugs affecting the activity of selected high-risk genes in AML.

## Materials and methods

### Data collection

Standardized data for AML (TCGA abbreviation: LAML) and normal blood were downloaded from the UCSC (https://xenabrowser.net/) and GTEx (https://gtexportal.org/) databases, respectively. Additionally, the mRNA expression profiles, mutation annotation data, copy number variation (CNV) data, and clinical metadata were obtained from the UCSC database. Samples with incomplete clinical data were excluded from further analysis. We also downloaded AML microarray gene expression datasets, GSE12417 (Metzeler et al., 2008), GSE37642 (Li et al., 2013b), and RNA sequencing datasets, GSE106291 (Herold et al., 2018), and GSE146173 (Bamopoulos et al., 2020) from the NCBI Gene Expression Omnibus (GEO) and Sequence Read Archive (https://www.ncbi.nlm.nih.gov/sra), respectively. The *combat* function of the *sva* package (Leek et al., 2012) was used to remove any batch effects between the TCGA and GTEx datasets. TCGA AML expression dataset was used as training cohort for the model construction, while the four GSE datasets were used as validation cohorts (details in Supplementary Table S1).

### Differentially expressed genes (DEGs)

The “*limma-voom*” algorithm (Law et al., 2014) was used to identify the DEGs between AML and normal control patients. The raw read count data across the samples were used as input for the differential expression analysis, and the genes with an *adjusted p*-value < 0.05 and logFC >1 or < −1 were identified as DEGs.

### Gene ontology (Go) and kyoto encyclopedia of genes and genomes (KEGG) pathway analyses

The GO and KEGG pathway enrichment analyses of the DEGs between normal controls and AML patients were performed using the “*clusterProfiler*” R package (Yu G et al., 2012) with default parameters. The enriched GO terms, including biological process (BP), cellular component (CC), and molecular function (MF) and KEGG pathways with an adjusted *p < 0.05* were considered significant.

### Cox regression analysis

We used the survival (v 3.5.7) package for performing Cox regression analysis to identify the high-risk genes in AML. The genes that had a *p-value* < 0.05 and a hazard ratio (HR) > 1 in TCGA dataset were shortlisted. Further, among these, we selected the ones that were also identified as risk factors (*p < 0.05* and HR > 1) in any two of the four GSE expression datasets analyzed.

### Gene set variation analysis (GSVA)

GSVA, a gene set enrichment method that estimates variation of pathway activity over a sample population in an unsupervised manner (Hanzelmann et al., 2013) was employed to identify distinct hallmark pathways between normal tissue and AML samples. Additionally, we utilized GSVA to ascertain distinct hallmark pathways between high- and low-risk AML subtypes.

### Protein-protein interaction network analysis

The STRING database (https://string-db.org/) (Bajpai AK et al., 2020; Szklarczyk et al., 2021) was used to analyze the protein-protein interactions (PPIs) among the selected risk factors with the correlation coefficient of more than 0.15. The database contains PPIs for multiple species that are based on various evidence, such as text mining, experiments, co-expression, neighborhood, gene fusion, and co-occurrence.

### Construction of a consensus prognostic model based on machine learning

To construct a consensus prognostic model with high accuracy and stability, we integrated 11 ML algorithms. These algorithms include Artificial Neural Network (ANN) (Bayne et al., 2012), Survival Random Forest (Survival RF) (Rigatti, 2017), Lasso (Tibshirani, 1997), Enet (Paszke et al., 2016), Supervised Principal Component Analysis (Supervised PCA) (Bair et al., 2006), Extreme Gradient Boosting (XGBOOST) (Hou et al., 2020), Stepwise Cox, Partial Least Squares Regression cox (plsRcox) (Geladi and Kowalski, 1986), Gradient Boosting Decision Tree (GBDT) (Friedman, 2001), Ridge (Hastie et al., 2009), and Survival Support Vector Machine (Survival SVM) (Zhang et al., 2017). In order to accomplish this, we calculated all possible combinations for the direction parameter of the algorithms individually, as well as by combining different algorithms in pairs. Furthermore, some ML algorithms have different predictive effects upon changing the parameters. For example, the ANN algorithm with different number of hidden layers between the input and output layers will have different predictive effects. These models were combined with different sets of parameters. Thus, a total of 137 models were obtained. A brief detail about the ML algorithms and the rationale behind selecting them in the current study is provided below.

ANN is a model that imitates the structure and function of biological neural networks, commonly used in the field of machine learning. We use ANN to construct prognostic models because it is a powerful nonlinear model that can learn and understand the complex nonlinear relationships in medical data, thereby better predicting patient prognosis. RF is a classifier that contains multiple decision trees, which is an ensemble machine learning algorithm used for classification and regression problems. We adopt this method to build a prognosis model because it can reduce the risk of overfitting by integrating the results of multiple decision trees, and it can calculate the importance of feature variables in the prognosis model through the variable importance integration method, which helps us identify key regulatory genes. Lasso is a linear regression method that uses L1 regularization. It achieves parameter shrinkage and feature selection by introducing L1 regularization to the model coefficients, helping to reduce the complexity of the model and improve its generalization ability. We use Lasso to build prognostic models because the expression matrix typically contains a large number of features, but only a portion of them may be related to prognosis. By introducing L1 regularization to penalize feature coefficients, Lasso can shrink some coefficients to zero, thereby achieving the effect of feature selection, that Lasso can help identify key features related to prognosis, simplifying the model and improving prediction accuracy. Enet is a linear regression method that combines L1 regularization with L2 regularization. We used it for building prognostic models because it combines the advantages of Lasso and Ridge, which can maintain good group effects while also selecting key features. This helps improve the model’s generalization ability and enhance predictive performance. Supervised PCA is a machine learning algorithm that combines the ideas of principal component analysis (PCA) and supervised learning, preserving key feature information while reducing data dimensions. The reason we use supervised PCA to build prognosis models is because it can retain key feature variables discriminatively while reducing data dimensions, allowing us to identify key regulatory genes. Additionally, by removing interfering features, we can enhance the predictive accuracy of the model. Ridge is a linear regression method that uses L2 regularization. Similar to Lasso, Ridge also uses L2 regularization penalty, which means it cannot completely reduce the feature coefficients to zero. The reason we use the Ridge method to construct the prognosis model is because it can complement the shortcomings of Lasso L1 regularization and maintain group effects. Moreover, Ridge regression adds L2 regularization, hence, this method can have good generalization ability when facing complex clinical data. GBDT is an ensemble model machine learning algorithm of gradient boosting decision trees, which uses the method of gradient boosting to iteratively train the model. GBDT was used to build the prognostic models because it is a type of decision tree that can handle non-linear relationships well and can fit complex clinical data effectively. GBDT, as a decision tree, can automatically handle outliers and noise without the need for additional data preprocessing, so it has good robustness, which is very helpful for predicting complex and variable clinical data. XGboost is based on GBDT, however, it introduces regularization and multiple classifiers. XGboost was used to build prognostic models because it improves upon GBDT by introducing regularization to enhance the generalization ability of the model. Additionally, custom loss functions allow XGboost to adapt to various types of classifiers, including ranking and regression, which is beneficial for handling the complexity of clinical data. CoxBoost is a machine learning algorithm that combines the principles of gradient boosting and the Cox proportional hazards model. It utilizes gradient boosting, allowing it to handle non-linear relationships, which is a significant improvement over traditional Cox models that can only handle linear relationships. Additionally, because CoxBoost combines the Cox model, it can effectively adapt to clinical data. plsRcox is a method that combines partial least squares regression and the Cox proportional hazards model. It maps high-dimensional data to a low-dimensional space and applies Cox in the low-dimensional space to construct the proportional hazards model. We adopted the method of plsRCox to build a prognosis model because it can handle high-dimensional data and multicollinearity, helping to extract important information from clinical data and reduce the risk of model overfitting. Stepwise Cox is a statistical method used for survival analysis, which, in the case of multiple features, gradually determines the most significant features for influencing survival time or survival probability. We use Stepwise Cox to construct a prognostic model because it includes forward selection, backward selection, and stepwise regression, enabling the automatic selection of features most relevant to survival time or survival probability, thus building a simple and effective prognostic model.

Among the 11 algorithms, for ANN, XGboost, Enet, and Stepwise Cox different parameters were selected for model building. For ANN, we chose combinations of 5–15 hidden layer neurons because the prognosis model is not particularly complex. Generally, the number of hidden layer neurons is chosen to be around a dozen, so we selected 5–15 neurons to seek the optimal solution. In XGboost, we chose the maximum tree depth to be between 1–5 because the data for the single cancer prognosis model is limited. After referencing other prognosis models, we decided to select the optimal tree depth between 1–5. Enet is a combination algorithm of Lasso and Ridge. When alpha = 0, it is Ridge, and when alpha = 1, it is Lasso. Therefore, we chose alpha to be between 0.1–0.9 to seek the best alpha for predicting the prognosis based on different combinations of L1 regularization and L2 regularization. Stepwise Cox is a special Cox regression statistical method, which can choose to perform backward selection, starting from including all feature types, gradually removing one feature at a time, each time selecting a variable that significantly improves the model fitting after removal. It can also choose to perform forward selection, starting from a model that does not include any features, gradually adding one feature at a time, each time selecting a feature that significantly improves the model fitting, or a stepwise regression model that incorporates both modes, due to the complexity of clinical data, we separately used three different parameters of Stepwise Cox to construct prognostic models in order to pursue a better predictive effect.

### Survival analysis

We employed Support Vector Machine (SVM), Artificial Neural Network (ANN), Boruta, Random Forest (RF), and Extreme Gradient Boosting (XGBOOST) algorithms to individually predict the prognostic significance of the genes. The weights or importance values corresponding to the genes was obtained from each algorithm. Subsequently, the values were standardized, and the average value for each gene across the five algorithms was calculated. Then, the prognostic significance of each gene was evaluated using the final values derived from the normalization of z-scores across five algorithms.

### Immune infiltration analysis

The “*CIBERSORT*” algorithm version 1.1.0 (http://cibersort.stanford.edu/) (Chen et al., 2018) was used to evaluate the tumor infiltration of immune cells. The normalized gene expression values corresponding to the AML samples were used as input for the tool.

### miRNA co-expression and stemness feature analysis

The miRNA expression data corresponding to 188 AML patients were obtained from the TCGA database (Weinstein et al., 2013). The coexpression analysis between the miRNAs and genes was performed using *Pearson correlation* method. The associations with correlation coefficient values (*R*) > 0.4 or < −0.4 and with *p < 0.05* were considered significant. The positive and negative *R* values indicate the positive and negative correlations between the miRNAs and DHRGs, respectively.

The tumor dryness scores for DNAss (DNA methylation-based Stemness Scores), EREG-METHss (Epigenetically regulated DNA methylation-based Stemness Scores), DMPss (Differentially methylated probes-based Stemness Scores), ENHss (Enhancer Elements/DNAmethylation-based Stemness Scores), RNAss (RNA expression-based Stemness Scores), and EREG. EXPss (Epigenetically regulated RNA expression-based Stemness Scores) were calculated based on Malta et al.’s (2018) method (Malta et al., 2018) using mRNA expression and methylation signatures.

### Drug sensitivity prediction

Drug sensitivity data were obtained from the Genomics of Drug Sensitivity in Cancer database (https://www.cancerrxgene.org/) (Yang et al., 2013). The R package “*oncoPredict*” version 0.2 (Maeser et al., 2021) was used to download the IC50 values of each drug. Subsequently, correlation analysis was performed between drug sensitivity and the expression levels of selected genes. Additionally, the drug sensitivity differences between high- and low-risk groups were calculated.

### Molecular docking and drug prediction

We used software DOCK (v 6.10; https://dock.compbio.ucsf.edu/DOCK_6/) to predict the binding patterns of small molecules and protein complexes. Firstly, we downloaded the three dimensional protein structures of the selected genes from the Protein Data Bank database (https://www.rcsb.org/) (Berman et al., 2000). The proteins were pretreated with UCSF Chimera (v 1.15; https://www.cgl.ucsf.edu/chimera/) by adding hydrogen, assigning partial charges and protonation states, and energy minimization (Pettersen et al., 2004). Secondly, we selected a subset of spheres to represent the binding sites by using the largest cluster generated by *sphgen*. Thirdly, the chemical structures of the active drug compounds were collected using the ZINC15 database (https://zinc15.docking.org/) (Irwin et al., 2012). Finally, all compounds were docked into the binding sites of the target proteins and were visualized in UCSF chimera (v 1.14) and LigPlus (v 2019).

## Results

### Identification of DEGs between AML patients and normal control

**FIGURE 1:**
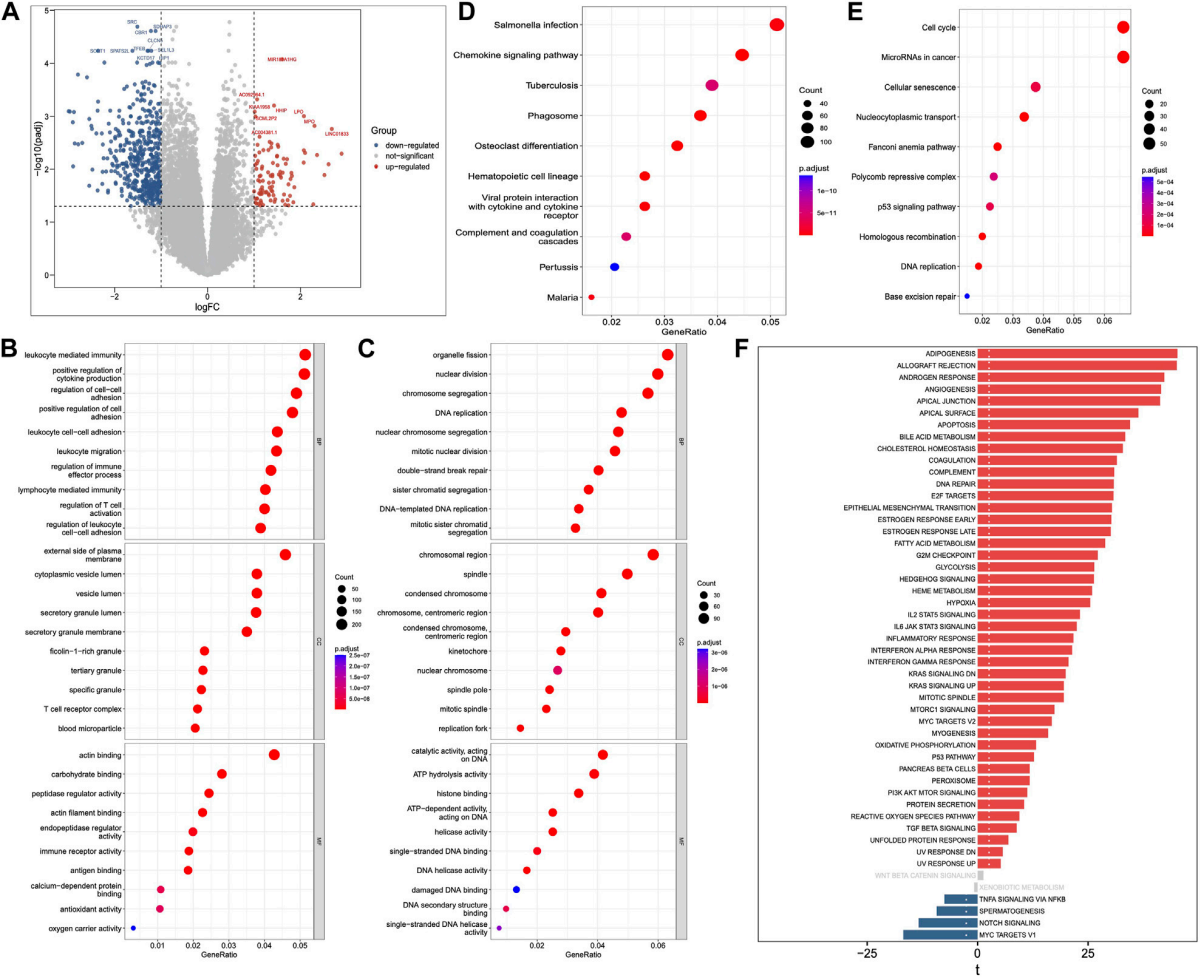
Differentially expressed genes (DEGs), Gene Ontology (GO), Kyoto Encyclopedia of Genes and Genomes (KEGG) pathway and Gene Set Variation Analysis (GSVA). **(A)** DEGs between AML and normal samples. **(B)** GO annotations of upregulated and **(C)** downregulated genes. **(D)** KEGG pathways of upregulated and **(E)** downregulated genes. **(F)** GSVA analysis of Hallmark pathways between AML and normal samples.

We identified a total of 5331 upregulated and 3230 downregulated genes in AML compared to normal blood samples using RNA-seq data obtained from TCGA and GTEx databases (Figure 1A; Supplementary Table S1). The functional enrichment analysis found 2123 GO annotations by the upregulated genes (Supplementary Table S2), and 339 annotations by the downregulated genes (Supplementary Table S3). Immune system related BPs and MFs were found to be significantly enriched by the upregulated genes (Figure 1B), whereas those related to DNA replication and repair were most enriched by the downregulated genes (Figure 1C). Furthermore, we found 154 KEGG pathways to be enriched by the upregulated genes (Supplementary Table S4), and 27 by the downregulated genes (Supplementary Table S5). Similar to the GO results, many of the top 10 upregulated pathways (Figure 1D) were related to immune response (e.g., chemokine signaling, hematopoietic cell lineage, and coagulation cascade). The downregulated pathways were related to cell cycle, DNA replication and repair, as shown in Figure 1E. Additionally, the GSVA results of the DEGs showed enrichment of various hallmark pathways associated with immune response, p53 signaling, cell cycle, DNA replication and repair, and signaling (Figure 1F).

### Identification of differential high-risk genes (DHRGs)

**FIGURE 2:**
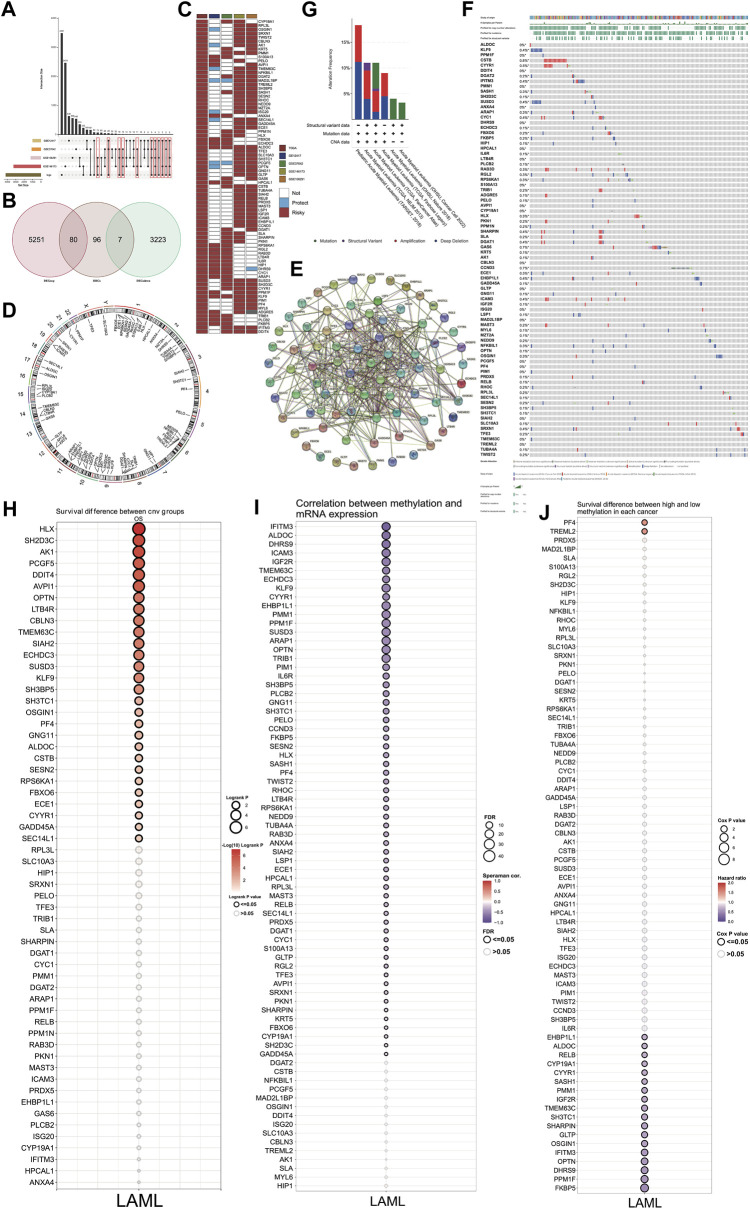
Identification and mutation of DHRGs. **(A)** Identification of high-risk genes common between TCGA and any two GSE datasets. **(B)** Venn plot showing the intersection of DEGs and high-risk genes. **(C)** Heatmap showing the HR and *p* values of DHRGs in the TCGA and four GSE datasets (“Risky”: HR > 1 and *p* < 0.05, “Protect”: HR < 1 and *p* < 0.05, “Not,” *p* < 0.05). **(D)** Chromosome circular plot showing the distribution of DHRGs on the chromosomes. **(E)** Protein-protein interaction (PPI) analysis of DHRGs. **(F)** Waterfall plot showing the frequency of mutations and copy number variations of DHRGs. **(G)** Alteration frequency of DHRGs in 6 different databases. **(H)** Survival difference between CNV group of DHRGs. **(I)** Correlation between methylation and RNA expression of DHRGs. **(J)** Survival difference between high and low methylation status of DHRGs.

The high-risk genes in AML were identified by Cox regression analysis of TCGA and four GSE datasets. The genes identified based on TCGA data had a *p-value* < 0.05 and HR > 1. Furthermore, among these, we selected the ones that were also high-risk factors (*p-value* < 0.05 and HR > 1) in any two of the four GSE datasets analyzed (Figure 2A). By comparing the high-risk genes with the DEGs, we found 80 genes that were high-risk factors in AML as well as upregulated in AML compared to normal control. These genes, henceforth referred to as differential high-risk genes (DHRGs) were considered for further analysis (Figures 2B, C). Figure 2C; Supplementary Figure S1 show the HR and *p-values* of the DHRGs across TCGA and GSE datasets. The chromosomal analysis of the 80 DHRGs showed that they are distributed across all 23 pairs of chromosomes except on 18 and Y chromosomes (Figure 2D).

To depict the functional significance of these genes, we constructed a PPI network using the STRING database. Our results showed that most of the 80 DHRGs interacted with each other indicating a close functional relationship among these proteins (Figure 2E). However, a few proteins including *FBXO6*, *ECE1*, *GLTP*, and *MAST3* were not part of the large network, while *MZT2A* had no interaction with any of the DHRGs.

### Genetic and epigenetic alterations in DHRGs and their effect on survival of AML patients

We used cBioPortal (http://www.cbioportal.org/) to analyze the genetic alterations associated with the DHRGs. A few of these genes had mutations, however the frequency was not high (Figure 2F). Furthermore, based on the data analyzed from 6 different datasets, including the complete Oregon Health & Science University (OHSU) AML cohorts (Tyner et al., 2018; Bottomly et al., 2022), three TCGA datasets (Ley et al., 2013; Tomczak et al., 2015; Hoadley et al., 2018) and the Therapeutically Applicable Research to Generate Effective Treatments (TARGET) AML initiative dataset (Bolouri et al., 2018), and we found that the alteration frequency in these genes ranged from 4% to 17% (Figure 2G). Together, these 6 datasets contained a total of 3239 samples from 2866 patients. While the OHSU datasets contributed to maximum number of samples (*n* = 1,614), the TARGET and TCGA (3 datasets together) contributed to 1,025 and 600 samples, respectively. We then focused on the effect of copy number variations (CNVs) and methylation status of the DHRGs on their RNA expression and survival of AML patients. The results revealed an association between the CNVs of 28 DHRGs and overall survival, with CNVs in *SH2D3C*, *HLX*, and *AK1* genes significantly affecting the survival of AML patients (Figure 2H). The correlation between methylation status and mRNA expression of the DHRGs revealed that the methylation in *TRIB1*, *OPTN*, *ARAP1*, *SUSD3*, *PPM1F*, *EHBP1L1*, *KLF9*, *IGF2R*, *ICAM3*, *ALDOC*, and *IFITM3* negatively regulated their mRNA expression (Figure 2I). Furthermore, we observed that the methylation of *PF4* and *TREML2* were high-risk factors with HR > 1 (Figure 2J), whereas methylation in 19 genes was found to be significantly associated with improved survival of AML patients (*p < 0.05* and HR < 1) (Figure 2J).

### Development of a robust consensus ML-driven signature

**FIGURE 3:**
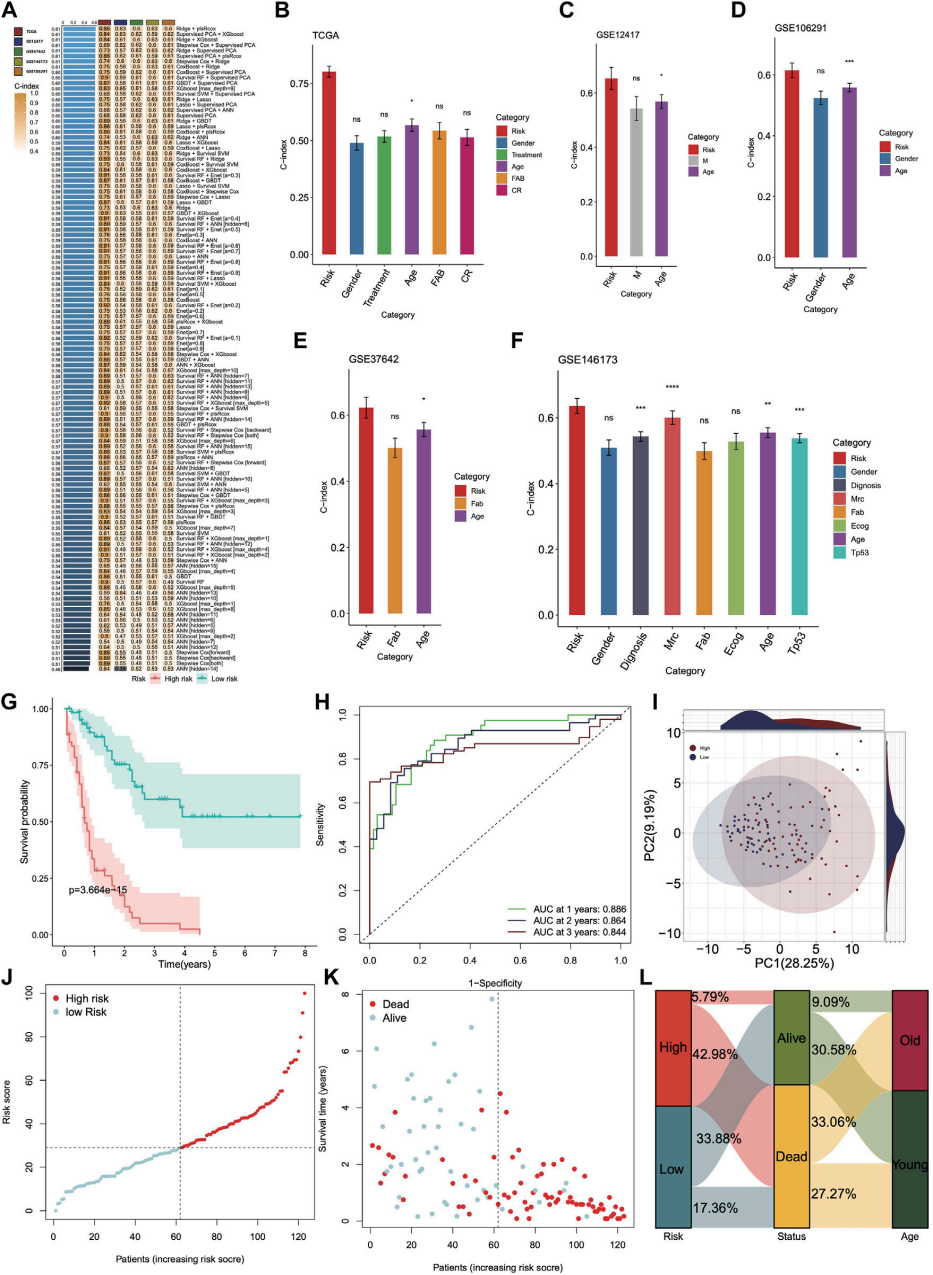
Construction and validation of the DHRGs-derived prognostic signatures via the machine learning-based integrative approach. **(A)** C-indices of the test and validation prognostic signature sets for each model. **(B–F)** The predictive performance of prognostic signature was compared with common clinical and molecular variables in the TCGA and four GSE datasets. **(G)** Survival curve of patients in high- and low-risk groups in the TCGA database. **(H)** ROC curve showing the AUC value of the model for different survival times. **(I)** PCA analysis showing the PC values of high- and low-risk groups. **(J,K)** Distribution of the risk score and survival status. **(L)** Sankey plot showing the proportion of surviving and deceased patients in high- and low-risk groups.

We used the DHRGs in an ensemble framework to perform a consensus ML-driven signature analysis. For the TCGA training cohort, we built consistent models using individual as well as combination of machine learning algorithms and calculated the C-index for each model. For the four GSE validation datasets, we calculated the C-index for each training model and then averaged it across the four datasets to assess the predictive power of all models (Figure 3A). C-index, or concordance index, is used to evaluate the predictive ability of the model. The c-index refers to the proportion of pairs in which the predicted results of patients are consistent with the actual results. Among the 137 models, the combination of Ridge and plsRcox algorithms maintained the highest mean C-index to build the final model. Furthermore, we compared the performance of DHRGs with other clinical and molecular variables in predicting prognosis. As shown in Figures 3B–F, DHRGs had distinctly superior accuracy compared to other variables, including gender, treatment, age, FAB stage, CR stage, M stage, and diagnosis.

To further evaluate the prognostic significance of DHRGs, we categorized the TCGA AML patients into high- and low-risk DHRG groups based on the median value. The Kaplan-Meier curve for the overall survival (OS) demonstrated that the low-risk DHRG group had significantly longer survival in the AML training cohort (Figures 3G, H). The AUCs for 1-, 2-, and 3-year OS were 0.886, 0.864, and 0.844, respectively. Additionally, to highlight the differences in the expression patterns of DHRGs, we performed principal component analysis (PCA) based on the DHRGs of the low- and high-risk groups. The scatter plot showed substantial differences in the expression patterns of DHRGs between the groups (Figure 3I). We also calculated the risk score and clinical status between the two groups (Figures 3J, K) and found that the high-risk group had a higher mortality rate (Figure 3L). The majority of the low-risk survival group is younger than 60 years old, while the majority of the high-risk death group is older than 60 years old (Figure 3L).

**FIGURE 4:**
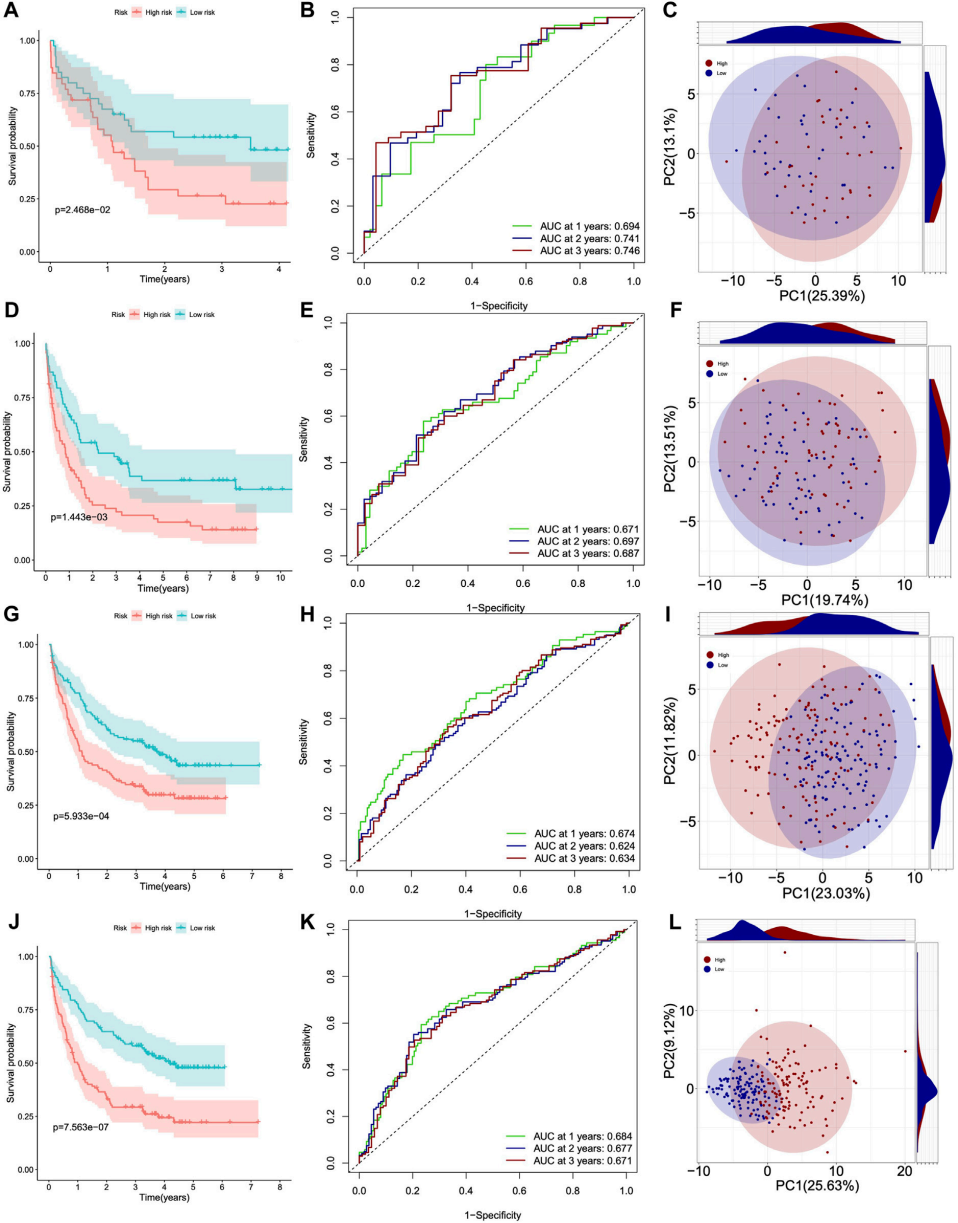
Survival curves, ROC curves and PCA analysis of four GSE datasets. **(A–C)** GSE12417. **(D–F)** GSE37642. **(G–I)** GSE106291. **(J–L)** GSE146173.

Next, we used four GSE datasets as test cohorts to further validate the feasibility of DHRGs for predicting AML prognosis. To maintain consistency with the training cohort, we determined the cutoff values for the low- and high-risk groups based on the median risk scores. The results of prognostic analysis were consistent with those of the training cohort. Kaplan-Meier survival curves showed that OS was poorer in the high-risk group than in the low-risk group (Figures 4A, D, G, J). The AUCs for OS were 0.694, 0.741, and 0.746 at 1, 2, and 3 years in GSE12417, 0.671, 0.697, and 0.681 at 1, 2, and 3 years in GSE37642, 0.674, 0.624, and 0.634 at 1, 2, and 3 years in GSE106291, and 0.684, 0.677, and 0.671 at 1, 2, and 3 years in GSE146173, respectively (Figures 4B, E, H, K). These relatively lower AUC values may be due to lower transcriptome differences between the high- and low-risk groups, higher intra-group variation, and RNA-seq batch effects. To investigate the batch effect, we performed PCA by combining the training and the four validation cohorts. There was a batch effect between the training and validation cohorts (Supplementary Figure S2). PCA analysis suggested that the expression pattern difference of DHRGs between the high- and low-risk groups was lower in the four test cohorts than in the TCGA cohorts (Figures 4C, F, I, L). We also calculated the risk score and clinical status between the two groups in the four test datasets, respectively (Supplementary Figures S3A, B, D, E, G, H, J, K), and our model predicted that over 70% of the high-risk group patients were deceased (Supplementary Figures S3C, F, I, L). We also found that the majority of the low-risk survival group is younger than 60 years old, while the majority of the high-risk death group is older than 60 years old (Supplementary Figures S3C, F, I, L).

Overall, Kaplan-Meier survival analysis, timeROC curve, and C-index of one training and four validation cohorts consistently indicated that DHRGs could accurately and robustly predict the prognosis of AML patients, suggesting that DHRGs may become an attractive tool for clinical practice.

### Predicting the importance of DHRGs for prognosis of AML patients

**FIGURE 5:**
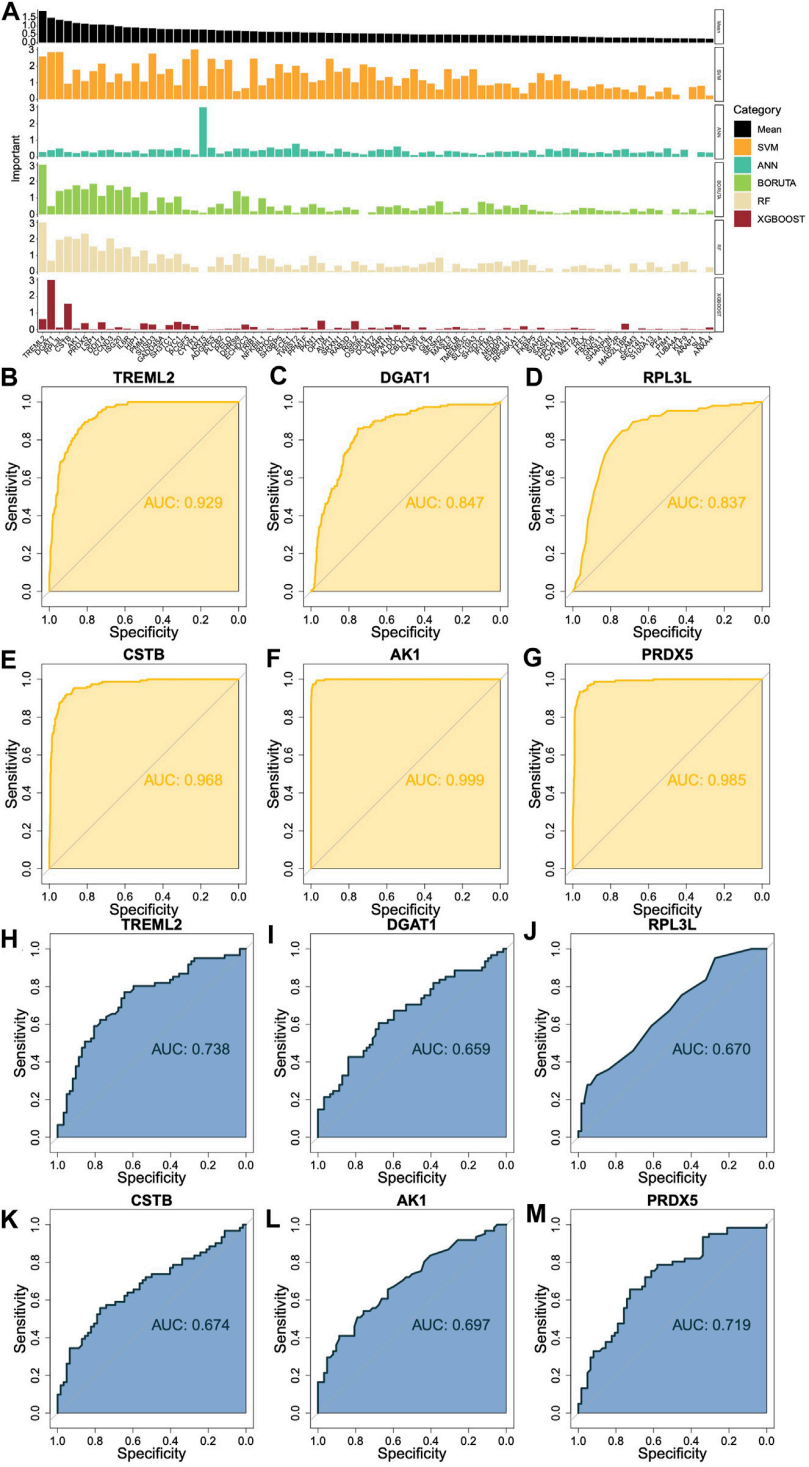
Prediction of the importance of DHRGs for prognosis of AML patients. **(A)** Prediction of the importance of DHRGs for prognosis of AML patients by five machine learning algorithms. **(B–G)** ROC curves to predict AML and normal conditions for *TREML2*, *DGAT1*, *RPL3L*, *CSTB*, *AK1*, and *PRDX5* respectively. **(H–M)** ROC curves to predict high and low-risk patients for *TREML2*, *DGAT1*, *RPL3L*, *CSTB*, *AK1*, and *PRDX5* respectively.

We utilized SVM (Supplementary Figures S4A, B), ANN (Supplementary Figures S4C, D), Boruta (Supplementary Figures S4E, F), RF (Supplementary Figures S4G, H), and XGBOOST (Supplementary Figure S4I) algorithms to individually predict the significance of DHRGs for prognosis. For each machine learning algorithm, we normalized the values according to the weights or important values of genes that affected survival. We identified the top 6 genes as *TREML2*, *DGAT1*, *RPL3L*, *CSTB*, *AK1*, and *PRDX5* (Figure 5A). Through ROC analysis, we observed high AUCs of 0.929, 0.847, 0.837, 0.968, 0.999, and 0.985, respectively (Figures 5B–G), which indicated that these genes could effectively predict normal and AML conditions. Furthermore, we conducted ROC analysis to predict high and low-risk patients using these 6 genes, resulting in AUCs of 0.738, 0.659, 0.670, 0.674, 0.697, and 0.719, respectively (Figures 5H–M). In comparison to predicting the high and low risks of AML patients, these genes demonstrated superior predictive ability for AML.

### Correlation and immune infiltration analysis

**FIGURE 6:**
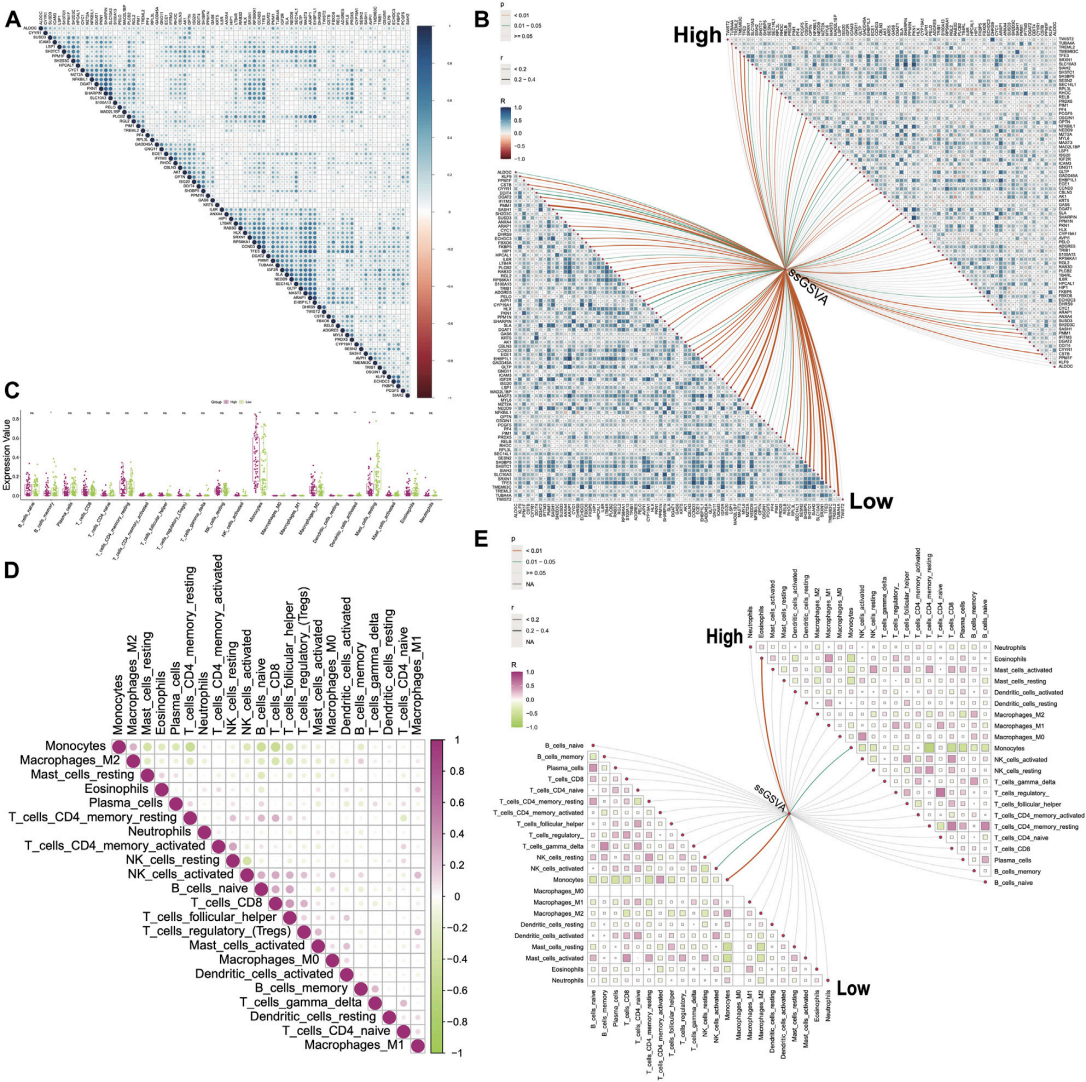
Correlations between genes and between immune cells. **(A)** Correlations between DHRGs in all group. **(B)** Correlations between DHRGs within as well as between high- and low-risk groups. **(C)** Differences in immune cell infiltration between high- and low-risk groups. **(D)** Correlations between immune cells in the ‘all’ group. **(E)** Correlations between immune cells as well as between immune cells and DHRGs-score in high- and low-risk groups. (ns: *p* > 0.05, *: *p* < 0.05, **: *p* < 0.01, ***: *p* < 0.001, ****: *p* < 0.0001).

We observed that 76 DHRGs showed significant differences between the high- and low-risk groups, with higher expressions in the high group compared to the low-risk group (Supplementary Figure S5). Subsequently, we calculated correlations among these DHRGs and identified positive correlations between most genes (Figure 6A; Supplementary Table S6). For instance, there was a strong positive correlation between *SHARPIN* and *SLC10A3* (R = 0.833), as well as between *ARAP1* and *EHBP1L1* (R = 0.855). In the high group, strong positive correlations were also found between *SHARPIN* and *SLC10A3* (R = 0.874), between *ARAP1* and *EHBP1L1* (R = 0.815), and between *SLC10A3* and *PKN1* (R = 0.853) (Figure 6B; Supplementary Table S7). Similarly, in the low group, we observed a strong positive correlation between *SHARPIN* and *SLC10A3* (R = 0.845), as well as between *ARAP1* and *EHBP1L1* (R = 0.894) (Figure 6B; Supplementary Table S8). In terms of the correlation coefficient between *SLC10A3* and *PKN1*, they were 0.639 and 0.737 for all samples and the low-risk group respectively. Interestingly, changes in the correlation patterns between genes were observed in the high and low-risk groups, indicating a decrease in correlation strength in the low group for some genes that showed strong correlation in the high group.

Next, we utilized the ssGSVA algorithm in the GSVA package to estimate the score of 80 DHRGs. We then calculated correlations between the expression of DHRGs and the estimated scores. In the low group, we identified several genes including *PMM1*, *MAST3*, *NEDD9*, *SH3TC1*, *SRXN1*, *TFE3*, and *TUBA4A* that showed positive correlations with the estimated score, with correlation coefficients exceeding 0.4 (Figure 6B; Supplementary Table S9). However, in the high group, the correlation coefficients between the DHRGs and the estimated score were less than 0.4 (Figure 6B; Supplementary Table S10).

The immune infiltration analysis revealed significant differences in monocytes, activated dendritic cells, and resting mast cells between the high and low groups (Figure 6C). We aimed to explore the changes in the linear relationships between immune cells in the all-, high-, and low-groups. In all-sample group, the strongest linear relationships were negative correlations between monocytes and memory resting CD4 T cells (R = −0.46), B cells (R = −0.43), and CD8 T cells (R = −0.48), respectively (Figure 6D; Supplementary Table S11). In the high group, monocytes exhibited negative correlations with plasma cells (R = −0.47), CD8 T cells (R = −0.54), memory resting CD4 T cells (R = −0.68), and eosinophils (R = −0.41) (Figure 6E; Supplementary Table S12). On the other hand, in the low group, monocytes were negatively correlated with plasma cells (R = −0.44), CD8 T cells (R = −0.41), memory resting CD4 T cells (R = −0.43), resting mast cells (R = −0.41), and activated mast cells (R = −0.48), while positively correlated with memory activated CD4 T cells (R = 0.41) (Figure 6E; Supplementary Table S13). Notably, changes in the correlation patterns between immune cells were observed in the high and low groups, with more prominent changes compared to those observed between genes.

Finally, we calculated correlations between DHRGs and immune cells and observed that in the low-risk group, nearly one-fourth of the genes positively regulated monocytes and negatively regulated resting mast cells (*p < 0.05*). However, in the high-risk group, the positive correlations between these genes and monocytes were weak, and the majority of these genes did not negatively correlate with mast cells (Supplementary Figure S6).

### miRNA coexpression and stemness features analysis

**FIGURE 7:**
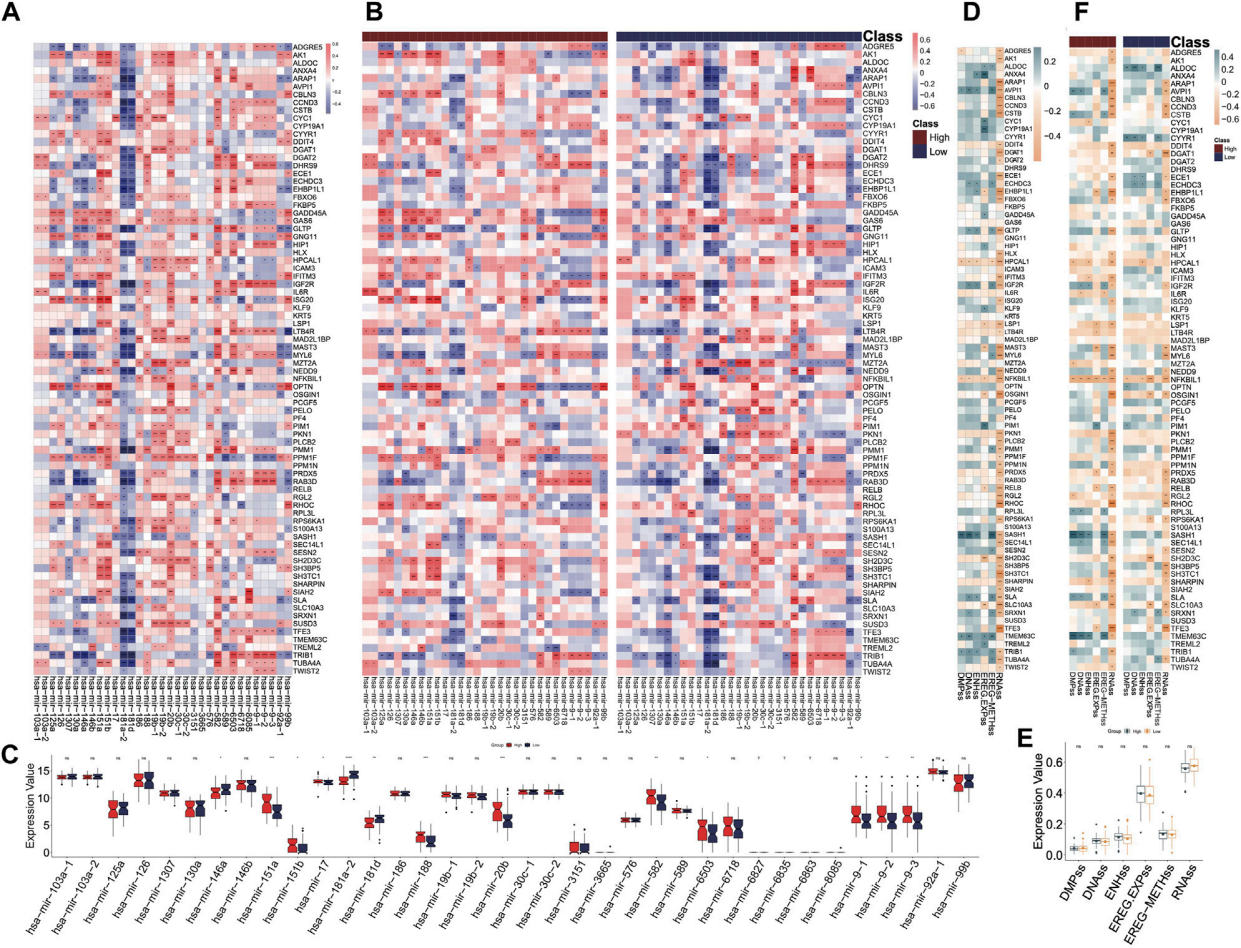
miRNA coexpression and stemness features analyses. **(A)** Correlations between miRNAs and DHRGs in the “all” group. **(B)** Correlations between miRNAs and DHRGs in high- and low-risk groups. **(C)** Differences in the expression of miRNAs between high- and low-risk groups. **(D)** Correlations between stemness features and DHRGs in the “all” group. **(E)** Differences in stemness features between high- and low-risk groups. **(F)** Correlations between stemness features and DHRGs in the high- and low-risk groups. (ns: *p* > 0.05, *: *p* < 0.05, **: *p* < 0.01, ***: *p* < 0.001, ****: *p* < 0.0001).

The correlations between all miRNAs and DHRGs in AML were analyzed, and the results showed that 33 miRNAs were significantly associated with DHRGs, with *R* > 0.4 or < −0.4 (Figure 7A). In the entire group, hsa-mir-181a-2 negatively regulated 21 genes (R < −0.4), hsa-mir-181d negatively regulated 16 genes (R < −0.4), and hsa-mir-582 positively regulated 12 genes (R > 0.4) (Figure 7A; Supplementary Table S14). However, in the high-risk group, hsa-mir-181a-2 and hsa-mir-181d negatively regulated only 7 and 10 genes, respectively (R < −0.4), and hsa-mir-582 positively regulated only 5 genes (R > 0.4). Additionally, hsa-mir-151a and hsa-mir-151b positively regulated more than 10 genes each (Figure 7B; Supplementary Table S15). In the low-risk group, hsa-mir-181a-2 and hsa-mir-181d negatively regulated 33 and 23 genes respectively (R < −0.4), and hsa-mir-582 positively regulated 21 genes (R > 0.4). Furthermore, hsa-mir-146a and hsa-mir-92a-1 negatively regulated 23 and 10 genes respectively (R < −0.4), while hsa-mir-6503 positively regulated 11 genes (R > 0.4) (Figure 7C; Supplementary Table S16).

Next, we calculated the correlations between DHRGs and stemness features. Almost half of the genes were negatively correlated with the indicator RNAss (Figure 7D). Furthermore, there were no differences in the six stemness features between the high- and low-risk groups (Figure 7E). Overall, the negative correlation between genes and RNAss decreased in both high- and low-risk groups (Figure 7F).

### Correlation of DHRGs with hallmark pathways

**FIGURE 8:**
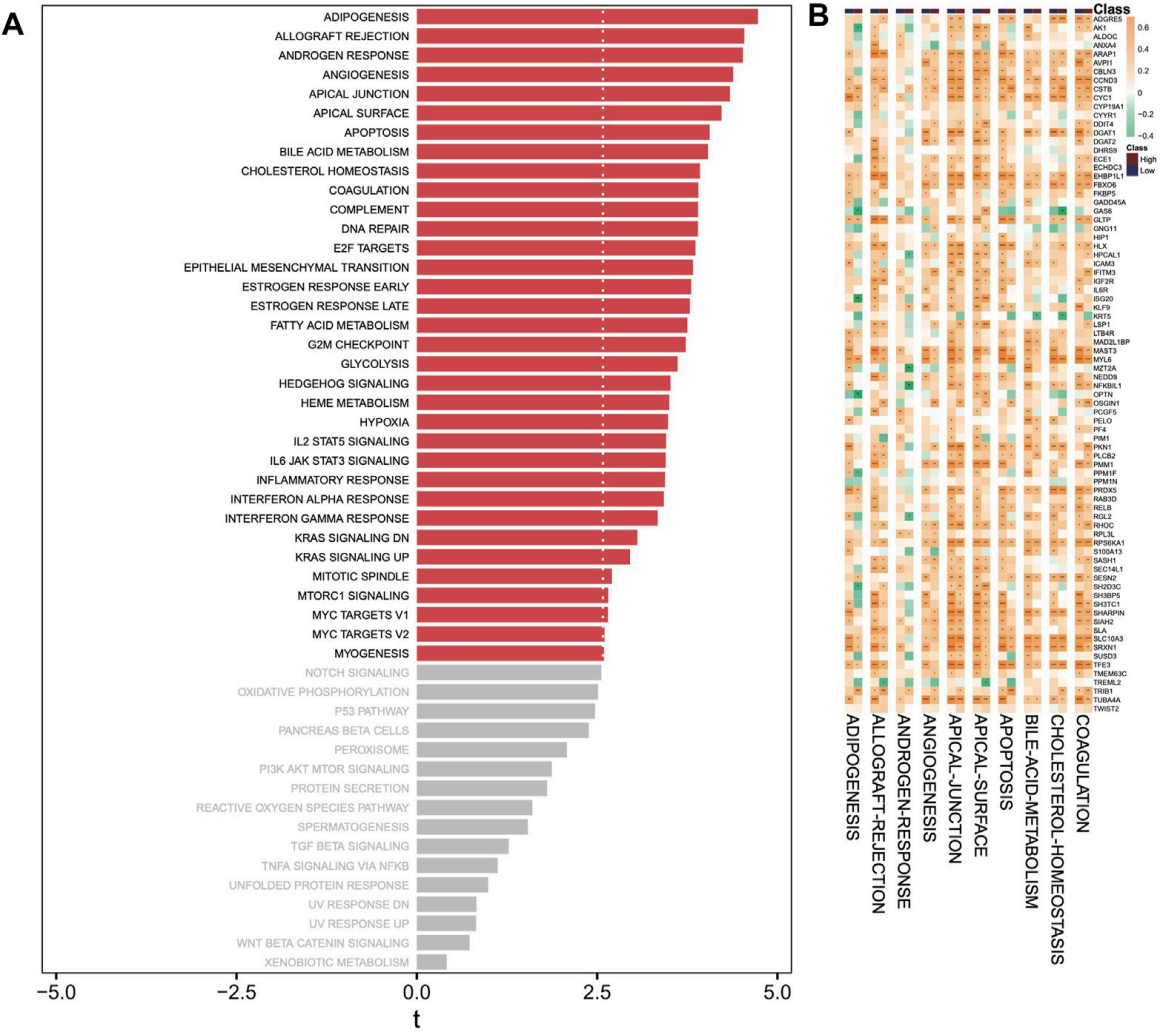
GSVA analysis. **(A)** Analysis of hallmark pathways between high- and low-risk groups. **(B)** Correlation analysis between top 10 hallmark pathways and DHRGs. (ns: *p* > 0.05, *: *p* < 0.05, **: *p* < 0.01, ***: *p* < 0.001, ****: *p* < 0.0001).

According to the hallmark pathways of GSVA analysis, we observed that adipogenesis, allograft rejection, androgen response, angiogenesis, and apical junction were enriched in the high-risk group (Figure 8A). Our focus was on the correlations between the top 10 hallmark pathways and the DHRGs. Generally, the regulatory relationships of genes in the low-risk group on pathways were higher than those in the high-risk group (R > 0.4 or R < −0.4) (Figure 8B; Supplementary Table S17). In both the risk groups, apical junction was the most regulated pathway, with 32 and 23 genes regulating it in the low- and high-risk groups, respectively (R > 0.4 or R < −0.4). The pathway with the most significant regulatory change from the low to high-risk group was apical surface. In the low-risk group, 31 genes regulated this pathway, whereas in the high-risk group, only 12 genes regulated it (R > 0.4 or R < −0.4).

### Relationship between drug responses, DHRGs and immune cells

**FIGURE 9:**
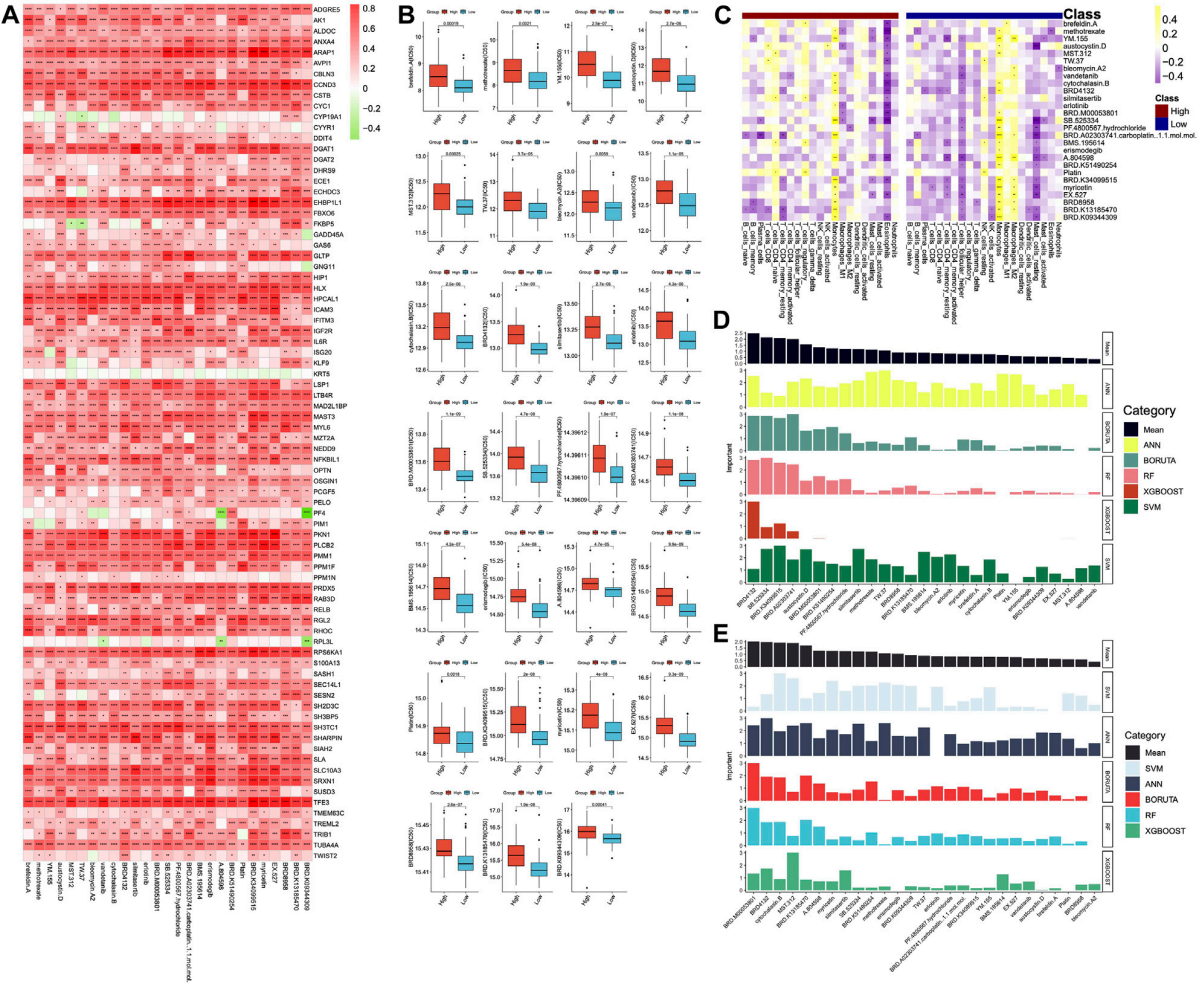
Prediction of drugs. **(A)** Correlations between DHRGs and predicted drug responses. **(B)** Differences in the sensitivity of the drugs between high- and low-risk groups. **(C)** Correlations between the immune cells and predicted drugs. **(D)** Prediction of the important drugs for DHRGs by five machine learning algorithms. **(E)** Prediction of the important drugs for prognosis of AML patients by five machine learning algorithms. (ns: *p* > 0.05, *: *p* < 0.05, **: *p* < 0.01, ***: *p* < 0.001, ****: *p* < 0.0001).

Furthermore, we utilized the *OncoPredict* package to predict the correlations between DHRGs and existing drug responses. The analysis revealed that 27 drugs had the potential to positively regulate the RNA expressions of fewer than 40 genes (Figure 9A). It was observed that the sensitivity of these drugs was significantly lower in the low-risk group (Figure 9B). Additionally, we examined the regulatory relationships of drugs on immune cells and discovered that 12 drugs positively regulated monocytes, while 14 drugs negatively regulated eosinophils in the high-risk group (*p < 0.01*). However, only 1 drug negatively regulated eosinophils in the low-risk group (*p < 0.01*) (Figure 9C). Furthermore, it was observed that 15 drugs positively regulated monocytes, and 8 drugs negatively regulated resting mast cells in the low-risk group (*p < 0.01*) (Figure 9C).

To estimate the importance of these 27 drugs that affect DHRGs and prognosis, we employed 5 machine learning algorithms. The analysis revealed that the top 5 drugs affecting DHRGs score were BRD4132, SB.525334, BRD.K34099515, BRD.A02303741, carboplatin, and austocystin. D (Figure 9D). Subsequently, the top 5 drugs that potentially impacted prognosis were BRD.M00053801, BRD4132, cytochalasin.B, MST.312, and BRD.K13185470 (Figure 9E).

### Binding of drug molecules with DHRGs

**FIGURE 10:**
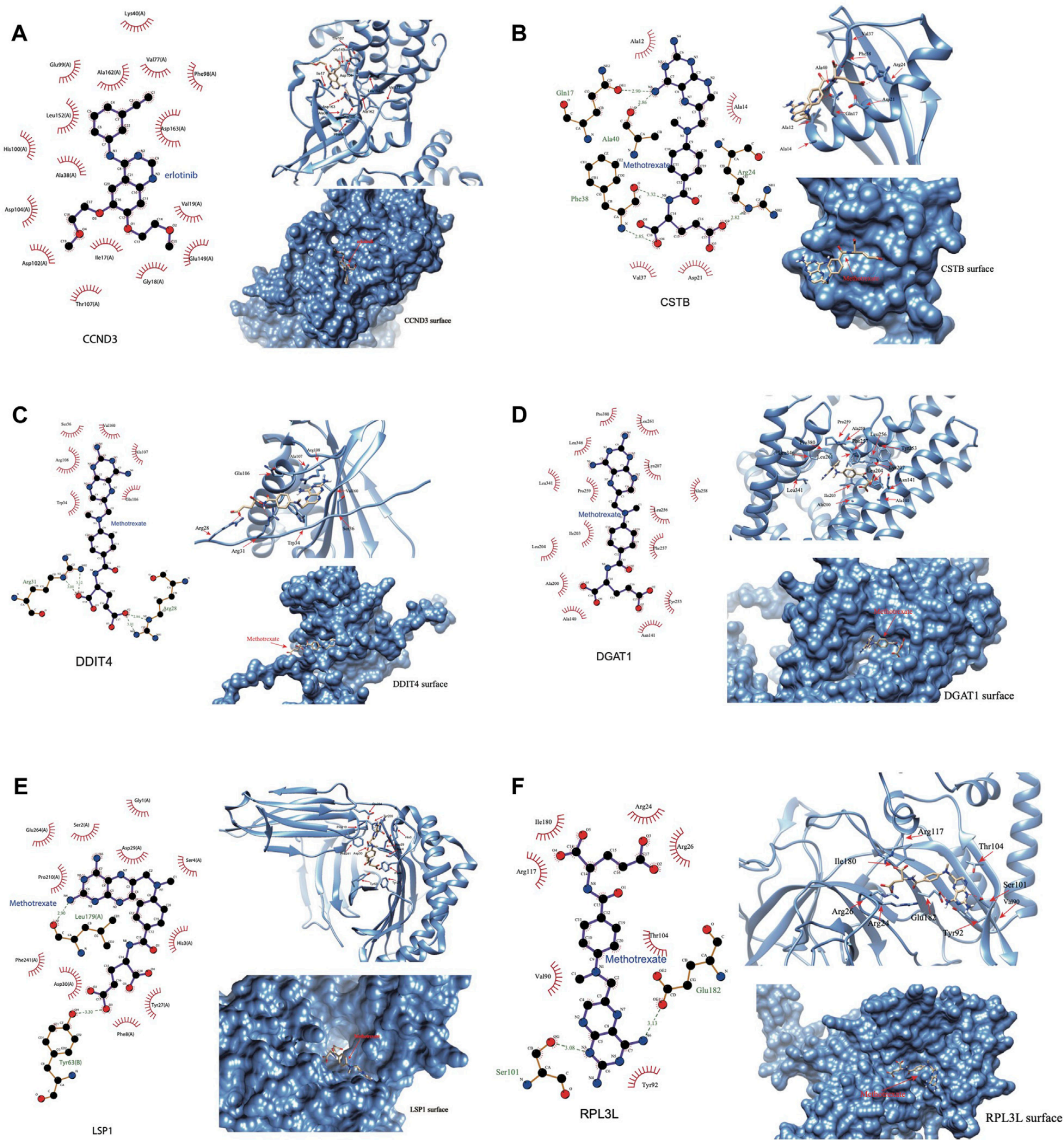
Binding of the drug molecules with DHRGs. **(A)** CCND3. **(B)** CSTB. **(C)** DDIT4. **(D)** DGAT1. **(E)** LSP1. **(F)** RPL3L.

We were able to download the chemical structure of only 6 active compounds, namely, methotrexate, vandetanib, silmitasertib, erlotinib, erismodegib, and myricetin from the ZINC15 database. Subsequently, we selected 6 genes to demonstrate the binding modes between these genes and the drugs (Figures 10A–F). Methotrexate exhibited the strongest binding with CCND3 as indicated by the docking score. According to the 2D and 3D link graphs, methotrexate displayed stronger interactions with His95 and Lys22 amino acids of CCND3. Additionally, a pocket was identified on the surface of CCND3 protein molecule, allowing methotrexate to interact with it, leading to a relatively stable complex (Figure 10A). In case of CSTB, methotrexate interacted with Gln17, Ala40, Phe38, and Arg24 amino acids. Although there was no pocket on the surface of CSTB protein, the binding remained relatively stable due to the significant number of hydrogen bonds formed between the small molecule and the protein (Figure 10B). Furthermore, methotrexate interacted with Arg31 and Arg28 amino acids of DDIT4 (Figure 10C), whereas it bound to DGAT1 through the pocket located on its surface (Figure 10D). In case of LSP1, vandetanib interacted with Thr20 and adhered to the pocket on the protein surface (Figure 10E). RPL3L interacted with methotrexate through Ser101 and Arg28 amino acids (Figure 10F). The binding patterns of the 6 genes with all 6 drugs has been shown in Supplementary Figure S7.

## Discussion

The current study identified 80 DHRGs in AML based on differential expression analysis between the disease and control groups and Cox regression analysis of RNA-seq and microarray transcriptomic data. The DHRGs were further explored through PPI analysis, genetic and epigenetic alterations, miRNA-coexpression, immune infiltration, drug sensitivity and survival analysis to establish their importance in AML pathophysiology. Furthermore, 11 ML algorithms were used to build prognostic models and the one constructed based on the combination of Ridge and plsRcox algorithms was selected as the best model with highest mean C-index.

The DHRGs were identified based on two different types of datasets (microarray and RNA-seq) having a Cox regression *p-value* < 0.05 and HR > 1. Interestingly, the chromosomal analysis of these genes revealed their distribution across all 23 chromosomes, with chromosomes (Chr) 1, 6, 8, 9, 10, and 11 harboring comparatively higher number of genes. The abnormalities in Chr 1 have been reported to be most frequent in myeloid malignancies (Caramazza et al., 2010). Furthermore, decades of AML research have discovered several novel anomalies, including insertions, deletions, and translocations in Chr 1 (Coupland et al., 2002; Caramazza et al., 2010). Of note, certain regions, especially 1q21-1q32 and 1p11-13, might harbor pathogenetically relevant genes (Caramazza et al., 2010). Trisomy 8 is one of the most frequent cytogenetic alterations in AML (10%–15% cases) (Hemsing et al., 2019). Although rare, the deletion of the long arm of Chr 9 (del9q) is considered as an intermediate risk factor for AML, and is characterized by frequent mutations of *DNMT3A*, *WT1*, and *NPM1* genes (Dohner et al., 2010; Herold et al., 2017). The Chr 6; 9 translocation has been reported to be associated with specific subtype of leukemia (Lindern et al., 1990; Lindern et al., 1992). Translocation between Chr 10; 11 has been shown to result in the fusion of *MLL*-*MLLT10* by a significant proportion of studies (Beverloo et al., 1995; Berger et al., 1996; Van Limbergen et al., 2002), although fusion of other transcripts has also been reported in a few cases (Dreyling et al., 1998; Van Limbergen et al., 2002).

Association between CNVs and survival of AML patients indicated *SH2D3C*, *HLX*, and *AK1* genes to play key roles. *SH2D3C* (SH2 Domain Containing 3C) encodes an adaptor protein and is a member of a cytoplasmic protein family that is involved in cell migration. This gene has been reported to be hypomethylated in acute lymphoblastic leukemia (ALL), the most common childhood blood cancer (Navarrete-Meneses and Perez-Ver, 2017), while a recent study has identified *SH2D3C* as a prognostic biomarker of tumor progression and immune evasion for lung cancer (Yeh et al., 2021). *HLX* (H2.0 Like Homeobox), a highly expressed gene in bone marrow enables sequence-specific DNA binding activity and predicted to be involved in the regulation of T-helper cell differentiation. A study by Kawahara et al., demonstrated its role in early hematopoiesis and induction of AML in rodent models and humans (Kawahara et al., 2012). Overexpression of *HLX* has been shown to downregulate the genes involved in electron transport chain and upregulate PPARδ levels as well as activate AMPK pathway (Piragyte et al., 2018). High expression of *AK1* (Adenylate Kinase 1) has been shown to correlate with poor prognosis of AML patients undergoing chemotherapy, suggesting that it can be used as an independent factor for treatment selection (Qin et al., 2020). Methylation of the DHRGs significantly affected the survival of AML patients by either negatively or positively regulating the mRNA expression. Aberrant DNA methylation has been reported as a hallmark of AML, and the methylated gene sets could be used as biomarkers for therapeutic decision making and disease prognosis (Carmona et al., 2016; Li et al., 2016). DNA methylation possibly prevents activation of hypoxia-responsive genes, while itself is known to be influenced by hypoxia, which alters metabolic pathways, transcriptional regulation of epigenetic modulators, and affects the activity of epigenetic modifiers, suggesting a bidirectional relationship between epigenetic regulation and hypoxia in AML (Humphries et al., 2023). In our study, *PF4* and *TREML2* were identified as high-risk factors. *PF4* (Platelet Factor 4) encodes a member of the CXC chemokine family, serum levels of which can be used as potential markers for monitoring the disease and assessing the clinical outcomes in AML (Humphries et al., 2023). Furthermore, reduced expression of *PF4* has been shown to promote the proliferations of human hematopoietic stem and progenitor cells (Meier-Abt et al., 2021). *TREML2* (Triggering Receptor Expressed On Myeloid Cells Like 2) is a cell surface receptor that may play a role in innate and adaptive immune response. In a study by Zhao et al., *TREML2* was found to be significantly associated with prognosis and was used along with five other genes to construct model equations for AML risk assessment (Zhao et al., 2018). However, further experimental studies are required to establish its detailed functional role in AML.

Further, the DHRGs were used for developing a consensus model using multiple ML algorithms individually and in combination. Out of more than 130 models tested by us, the combination of Ridge and plsRcox resulted in highest accuracy and were used for building the final model. While both these algorithms have been used for cancer research, particularly for predicting therapy response, prognosis and identifying novel gene signatures, the usage of Ridge has been more common for AML (White et al., 2021; Tang et al., 2022; Wei et al., 2022; Chen et al., 2023; Liu et al., 2022). When using ML for predicting the important prognostic genes for AML, *TREML2* was one of the top 6 with high AUC of 0.9, further confirming its importance in hematological malignancies. The other five significant prognostic genes with high AUCs were *DGAT1*, *RPL3L*, *CSTB*, *AK1,* and *PRDX5*. *DGAT1* (DiacylGlycerol O-AcylTransferase 1) encodes a multipass transmembrane protein, which acts as a key metabolic enzyme and has been explored in a few cancers including AML by a few studies (He et al., 2021; Liu et al., 2022). A recent study demonstrated the involvement of *ROS/p38 MAPK/DGAT1* pathway in AML progression. The upregulation of *DGAT1* due to the synergistic effects of elevated reactive oxygen species levels and activated p38 MAPK signaling pathway promotes accumulation of lipid droplets, eventually enhancing lipid peroxidation in AML cells (Liu et al., 2022). The protein encoded by *CSTB* (Cystatin B) functions as an intracellular thiol protease inhibitor. Honnemyr et al., studied the constitutive protease release by human AML cells and detected the release of CSTB for most patients (Honnemyr et al., 2017). Furthermore, Aasebo et al., showed heterogeneity in the intracellular and released levels of *CSTB* in AML patients (Aasebo et al., 2018). *PRDX2* (Peroxiredoxin 2) plays an antioxidant protective role in cells and has been identified as a novel potential tumor suppressor gene in AML. The expression of PRDX2 at mRNA and protein level is reduced due to the epigenetic modifications in its promoter region (Agrawal-Singh et al., 2012). *RPL3L* (Ribosomal Protein L3 Like) has also been reported as an epigenetically silenced tumor suppressor in endometrial cancer (Takai et al., 2005), however its function in AML is yet to be explored. Thus, the genes identified in the current study might pave way for the development of novel diagnostic and treatment strategies for AML or other hematological malignancies. Furthermore, the ML approaches that have been used by us for identifying the high-risk genes could also be employed for identifying similar candidates for other patho-physiological conditions. In addition, we have shown that the combination of ML algorithms could predict better candidates than using them individually. This method of combining ML algorithms for identifying prognostic genes provides the advantages of both algorithms, while complementing the limitations of one another, and thus can be used for improving patient outcomes. Furthermore, the prognostic models can be standardized for specific group of patients using their gene expression patterns and can be used for discovering candidate/prognostic genes specific to a subgroup of patients. There have been several reports promoting the use of AI-ML techniques in personalized medicine (Schork, 2019; Peng et al., 2021; Sebastiani et al., 2022). Thus, our study could aid in personalized medicine of hematological malignancies.

Immune infiltration and correlation analysis indicated differences in the immune cell abundance between high and low-risk AML groups that were predicted based on the expression pattern of DHRGs. Our results indicated that the correlations of the DHRGs with immune cell abundances vary between high- and low-risk AML groups. *SH3TC1* is one such gene that was significantly positively correlated with monocytes and negatively correlated with mast cells in the low-risk group, whereas no such correlations were observed in the high-risk group. A study by Langer et al., found that *SH3TC1* interacts with *MN1*, a gene of prognostic significance for AML (Langer et al., 2009). However, we could not find studies focusing on this gene in the context of AML, thus making this a suitable candidate for further exploration. Some of the other interesting genes potentially differentiating immune cell infiltration between high- and low-risk groups include *ANXA4*, *HLX*, *IL6R*, *HIP1*, *CSTB*, *SLA*, and *TREML2*.

The miRNA-DHRG interaction analysis identified several miRNAs negatively regulating the mRNA expression of DHRGs in AML. The hsa-mir-181 miRNA family (i.e., hsa-mir-181a-2, hsa-mir-181d) was found to be important and affected the expression of DHRGs in AML patients as well as in the high- and low-risk groups. The miR-181 family has been identified as a high-risk factor in head and neck cancers and could distinguish the malignant tumor from normal samples (Nurul-Syakima et al., 2011). This miRNA family has also been implicated in regulating the differentiation of B and T cells, natural killer cells during normal hematopoiesis and has been linked to the pathophysiology and prognosis of AML (Su et al., 2015; Weng et al., 2015; Huang et al., 2016; Gao et al., 2018). According to a study by Gao et al., hsa-mir-181a-2 was predicted to significantly affect the survival time of AML patients (Gao et al., 2018). Both mir-181d and mir-181a have been reported to downregulate the expression of *PRKCD*, *CTDSPL* and *CAMKK1* in AML patients by Su and his colleagues (Su et al., 2015). Furthermore, inhibition of the expression of the miR-181 family partially reversed myeloid differentiation blockage not only in AML bone marrow (BM) blasts but also in a mouse model of AML (Su et al., 2015).

When we looked at the hallmark pathways that were enriched in AML risk groups, adipogenesis was found to be the most enriched pathway in the high-risk group. A recent study by Azadniv et al. (2020) compared bone marrow mesenchymal stromal cells (MSCs) from normal donor and AML patients and found that MSCs derived from AML patients have higher adipogenic potential and may impact the survival of leukemia progenitor cells. Furthermore, using *in vitro* and *in vivo* models, Zhang et al. (2022) discovered that AML-derived exosomes may in turn be partially responsible for the reprogramming of MSCs, resulting in their differentiation to adipocytes, through a metabolic shift from glycolysis to oxidative phosphorylation, indicating the existence of a complex interaction of leukemia cells with their microenvironment. Chemotherapy treatment has been shown to reduce the adipocyte content in AML patients, possibly by promoting the overexpression and secretion of GDF15 from bone marrow mononuclear cells (Liu et al., 2018). These studies strongly support the enrichment of adipogenesis in the high-risk AML group by our DHRGs and their interactions with the immune cells. When the relationship between the drugs and immune cells was investigated by us, we found that a considerable number of drugs positively regulate that infiltration of monocytes in both high- and low-risk AML groups.

Using ML algorithms, we identified top 5 drugs, including carboplatin (also known as cisplatin) and austocystin-D that may significantly affect the DHRGs in AML. Carboplatin, an FDA approved drug, is used for the treatment of various cancers and has also been effective against AML. A clinical trial by Bassan et al. (1998) showed that combination of carboplatin, granulocyte colony-stimulating factor, high-dose cytarabine on alternate days and mitoxantrone/idarubicin is well tolerated, and exerted a significant activity in high-risk AML (Bassan et al., 1998). Austocystin D is an organic heteropentacyclic compound isolated from *Aspergillus* and *Aspergillus ustus* and possesses cytotoxic and anti-tumor activity through its selective activation by cytochrome P450 enzymes, leading to the induction of DNA damage (Marks et al., 2011). Another study evaluated the anti-tumor activity of austocystin-D-loaded liposomes (AD-Ls) and suggested that AD-Ls increase the cure efficiency and decrease the side effects on other tissues as shown in animal models of liver cancer (Li et al., 2013a). Cui et al., identified *TLN1* as a poor-prognostic biomarker in AML and showed that this gene may be related to the resistance of austocystin-D and few other drugs in AML cells (Cui et al., 2022). The other top drugs that were identified include SB-525334, BRD-K34099515, BRD-A02303741, and BRD4132. SB-525334 (6-[2-tert-butyl-5-(6-methyl-pyridin-2-yl)-1H-imidazol-4-yl]-quinoxaline) has been identified as a selective inhibitor of the transforming growth factor-beta1 (TGFβ1) receptor (Grygielko et al., 2005). Heo et al. (2021) showed that SB525334 effectively attenuates TGF-β1-induced epithelial to mesenchymal transition (EMT) in human peritoneal mesothelial cells. However, its effect on AML is yet to be studied. Similarly, the function of the other top drugs remains to be investigated in the context of AML. The molecular docking analysis revealed interaction of methotrexate with important DHRGs, including *CCND3*, *CSTB*, and *DDIT4*, *RPL3L* and *DGAT1*. Methotrexate is an FDA approved drug that is used for treating severe psoriasis, rheumatoid arthritis, and certain types of cancers including leukemia and lymphoma (Weinblatt et al., 2013). The interactions of multiple DHRGs with methotrexate further supports the key roles of these genes in AML related pathophysiology. Vandetanib is a tyrosine kinase inhibitor that acts against several pathways implicated in malignancy (Carlomagno et al., 2002; Hennequin et al., 2002). Macy et al., have demonstrated that vandetanib mediates anti-leukemia activity via multiple mechanisms and interacts synergistically with DNA damaging agents (Macy et al., 2012). Silmitasertib, a casein kinase 2 (CK2) inhibitor has been demonstrated as a drug for the treatment of human hematological malignancies (Chon et al., 2015). It is interesting to note that silmitasertib was the first drug that entered into clinical trials for the treatment of both hematological malignancies and solid tumors (D'Amore et al., 2020). Erlotinib has been shown to be effective against FLT3-ITD mutant AML and has the ability to overcome intratumoral heterogeneity via targeting FLT3 and Lyn (Cao et al., 2020). A pilot phase II study by Abou Dalle et al. (2018) has however shown that as a single agent it has limited clinical efficacy in patients with relapsed/refractory AML. Erismodegib is under clinical trials for patients with AML, in combination with other chemotherapies (Yu et al., 2020). It has been shown to target the Hedgehog signaling pathway (Tibes et al., 2015). Myricetin is a polyhydroxy flavonol found in a several types of plants and plays a significant role in cancer prevention via inhibiting the inflammatory markers, such as inducible nitric oxide synthase (iNOS) and cyclooxygenase-2 (Cox-2) (Rahmani et al., 2023). Moreover, myricetin increases the chemotherapeutic potential of other anticancer drugs through modulation of cell signaling activities (Rahmani et al., 2023).

The current study used extensive bioinformatics along with 11 ML algorithms to identify the hub genes in AML and predict their prognostic value. However, there are a few limitations. In the current study, we tested a total of 137 ML models by using pair-wise combinations to predict the prognostic value of the identified candidates and found the combination of Ridge and plsRcox to be the best model. However, the predictive ability of this combination needs to be validated with larger number of datasets as well as in other subtypes of leukemia/AML or disease conditions. Additionally, knock-in/-out animal models can be used to confirm the findings of the current study and annotate the functional significance of the candidate genes. Furthermore, the generalizability of the findings across diverse populations may need to be investigated in future, owing to the lack of population specific AML cohorts. Furthermore, it is possible that the sample heterogeneity may potentially impact the model predictions, which could be assessed in future studies.

## Conclusion

The current study used ML algorithms and various bioinformatics approaches to identify high-risk genes associated with AML (DHRGs). The expression pattern of DHRGs was able to successfully classify the AML samples into high- and low-risk groups. Genetic and epigenetic alterations helped in gaining better understanding of their regulation. Immune infiltration and survival analysis demonstrated the significance of DHRGs as prognostic indicators. Drug sensitivity and molecular docking studies revealed drugs with potential effect and genes that could be used a therapeutic drug target for inhibiting the growth and progression of AML. Further studies including experimental validations are required to select a few important candidates for detailed study of their functional roles in AML pathophysiology.

## Data Availability

The original contributions presented in the study are included in the article/Supplementary Material, further inquiries can be directed to the corresponding authors.
