# Supplementary material for: Identification of hub genes and potential molecular mechanisms related to drug sensitivity in acute myeloid leukemia based on machine learning
Source: Front Pharmacol. 2024 Apr 8;15:1359832. doi: 10.3389/fphar.2024.1359832 (PMC11033397; doi:10.3389/fphar.2024.1359832)
Supplement: Supplementary file 1 [file DataSheet1.zip › Additional files/Supplementary material.DOCX]

**Supplementary Figure 1.** HR and p values of DHRGs in the TCGA and four GSE datasets.


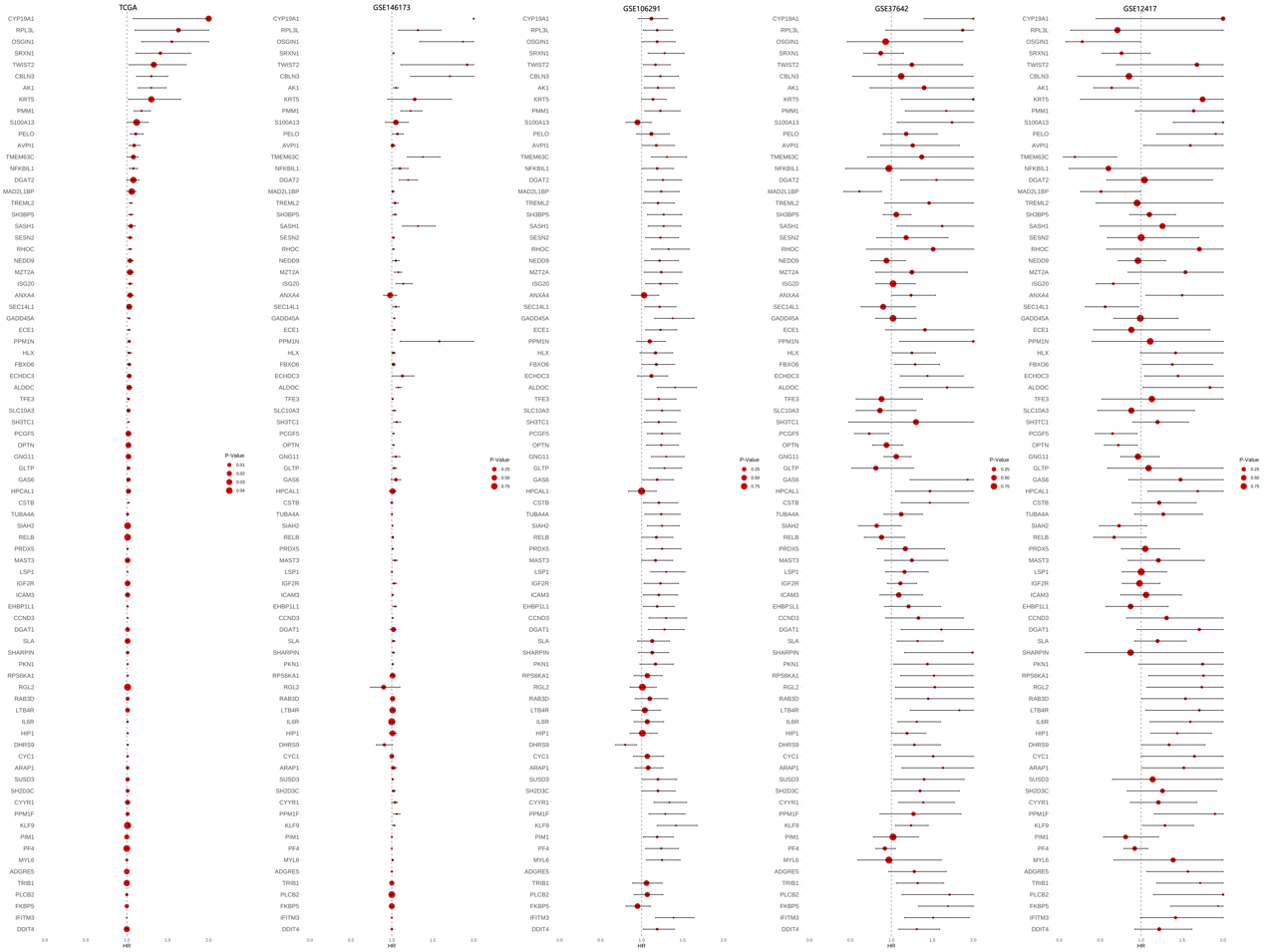


**Supplementary Figure 2.** PCA analysis of TCGA and the four GSE datasets.


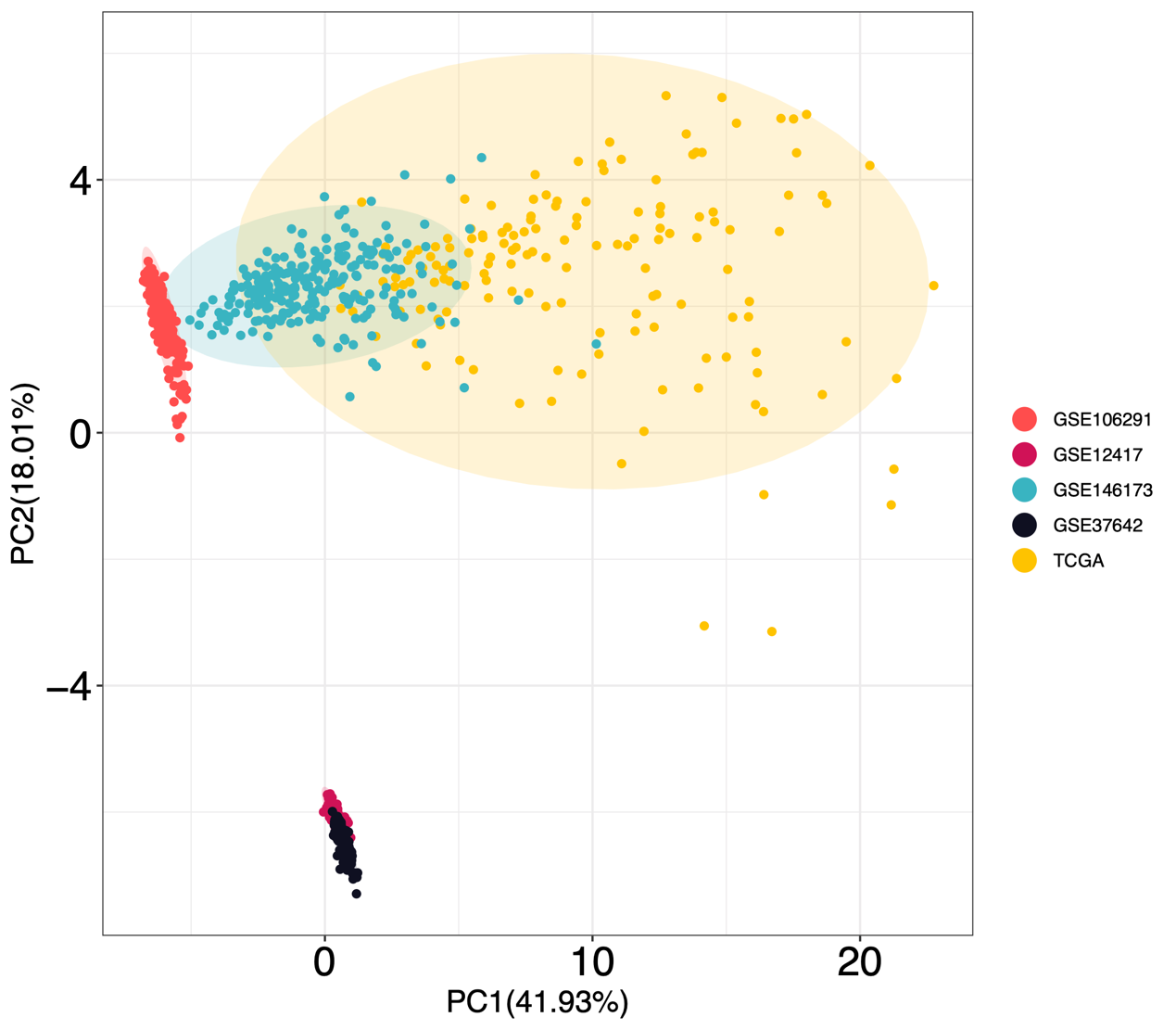


**Supplementary Figure 3.** The risk score, survival status, and survival ratio of high- and low-risk AML patients in the four GSE datasets. (A-C) GSE12417. (D-F) GSE37642. (G-I) GSE106291. (J-L) GSE146173.


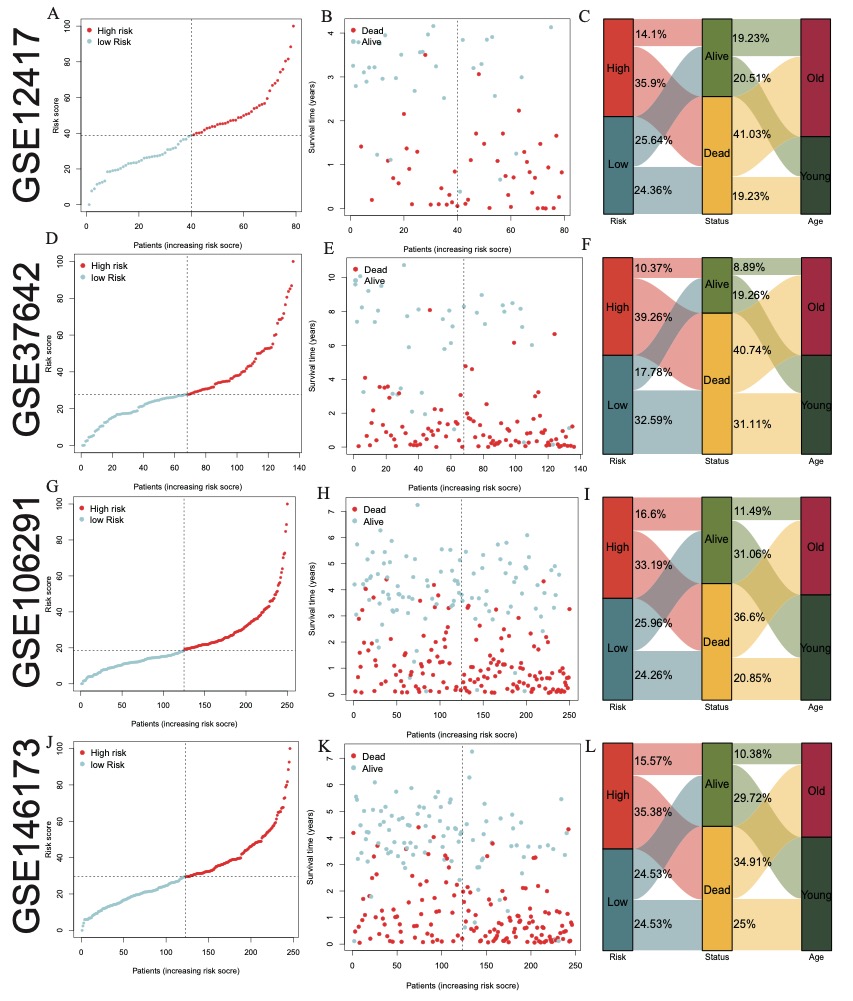


**Supplementary Figure 4. Results of five machine learning methods.** (A-B) SVM. (C-D) ANN. (E-F) Boruta. (G-H) RF. (I) XGboost.


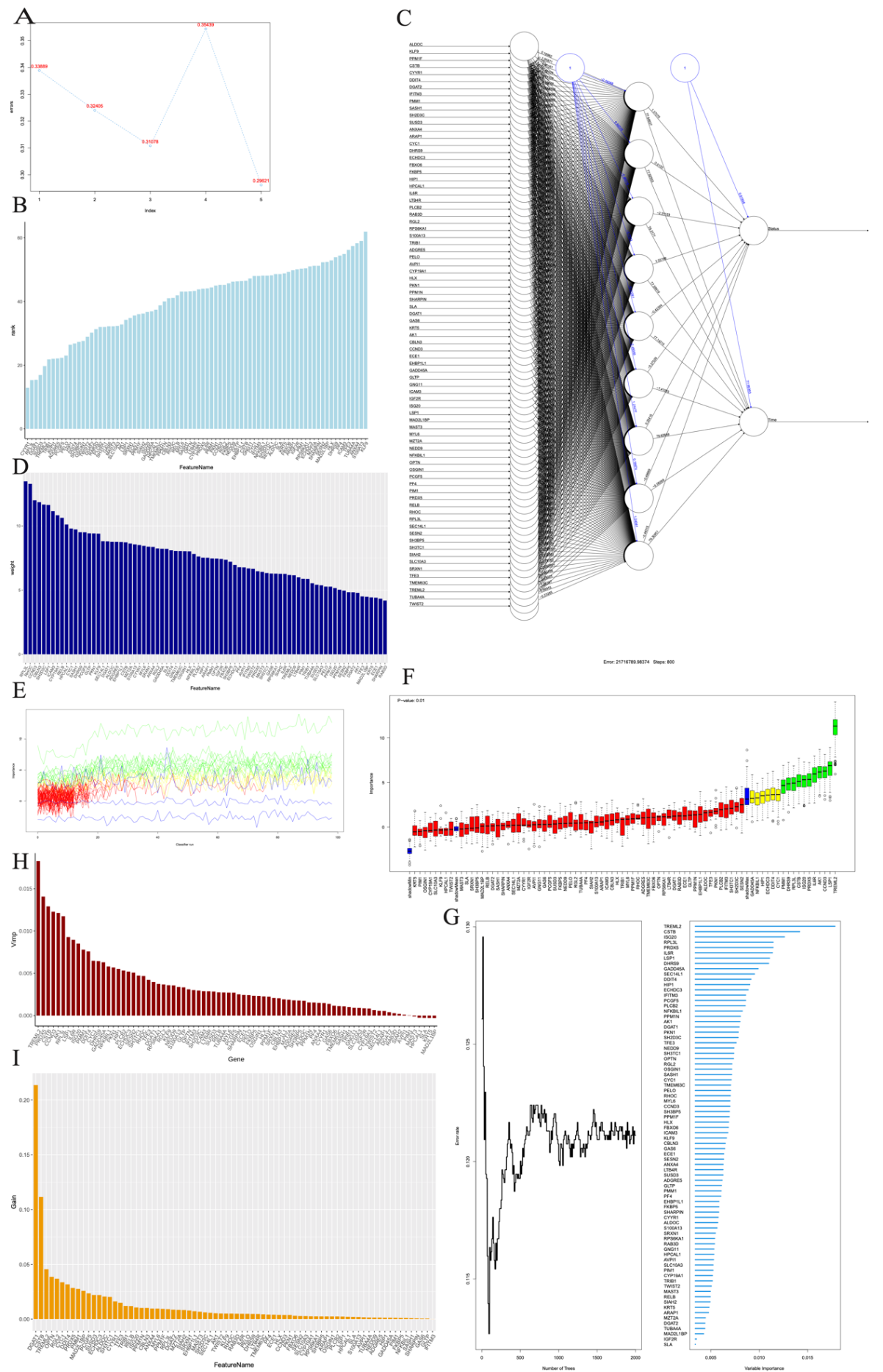


**Supplementary Figure 5. Differential expression of DHRGs between high- and low-risk AML groups.**


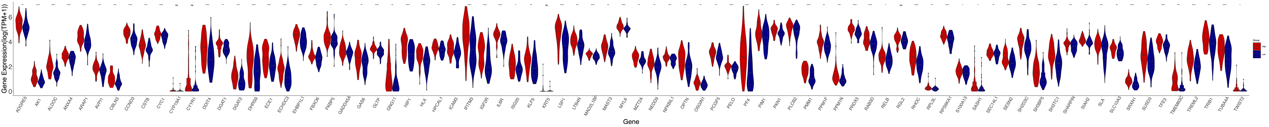


**Supplementary Figure 6. Correlations between immune cells and DHRGs in high- and low-risk AML groups.**


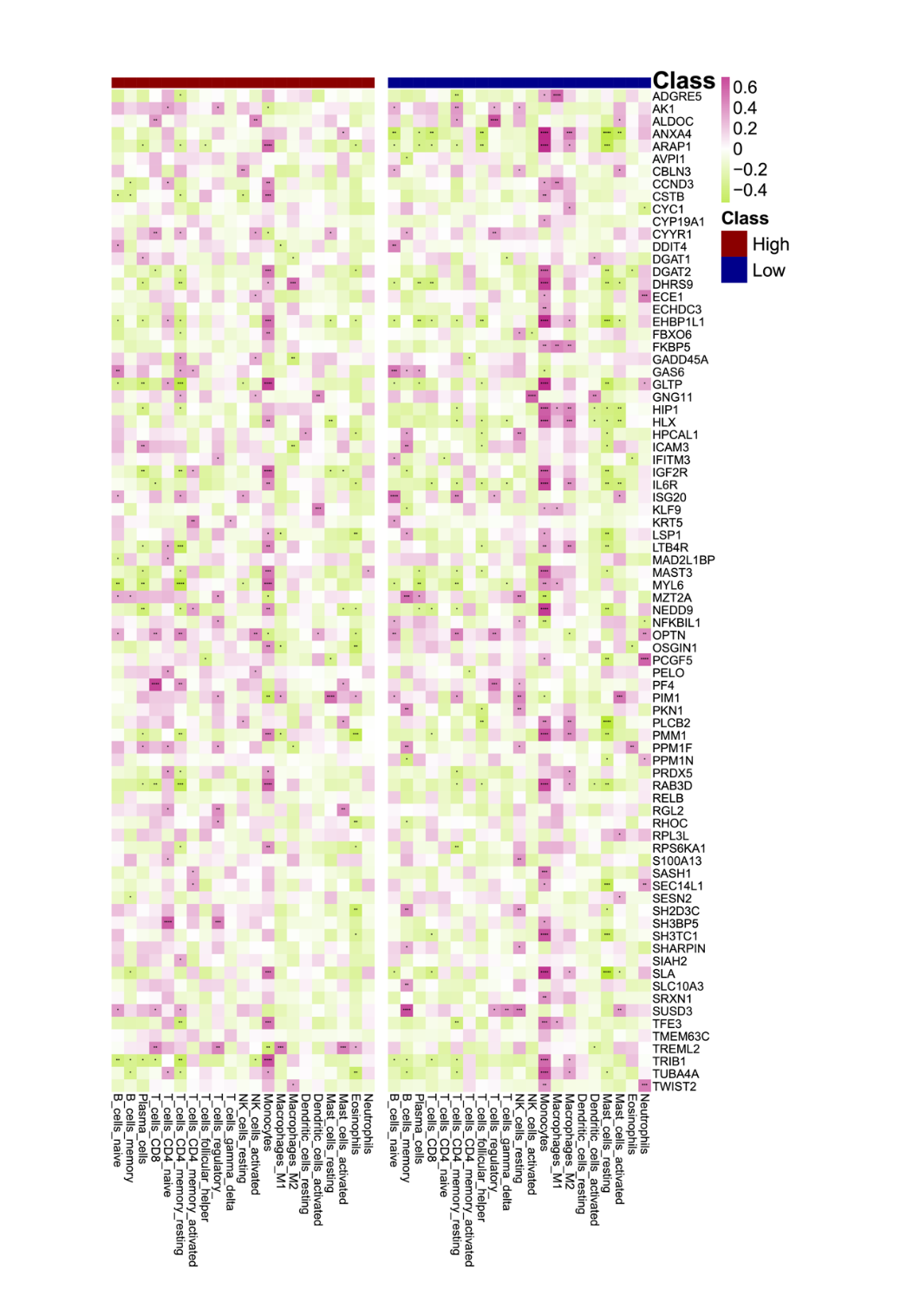


**Supplementary Figure 7. Binding of the drug molecules of six genes.**

**Supplementary Table 1.** Summary of datasets used as training and validation cohorts.

**Supplementary Table 2.** Differentially expressed genes between AML and normal control patients.

**Supplementary Table 3.** GO annotations by the upregulated genes in AML.

**Supplementary Table 4.** GO annotations by the downregulated genes in AML.

**Supplementary Table 5.** KEGG pathways by the upregulated genes in AML.

**Supplementary Table 6** KEGG pathways by the downregulated genes in AML.

**Supplementary Table 7.** Correlation coefficients between DHRGs in all AML patients.

**Supplementary Table 8** Correlation coefficients between DHRGs in the high-risk group.

**Supplementary Table 9.** Correlation coefficients between DHRGs in the low-risk group.

**Supplementary Table 10.** Correlation coefficients and *P*-values between DHRGs and DHRGs-score in high-risk group.

**Supplementary Table 11.** Correlation coefficients and *P*-values between DHRGs and DHRGs-score in low-risk group.

**Supplementary Table 12.** Correlation coefficients between immune cells in all AML patients.

**Supplementary Table 13.** Correlation coefficients between immune cells in high-risk group.

**Supplementary Table 14.** Correlation coefficients between immune cells in low-risk group.

**Supplementary Table 15.** Correlation coefficients between miRNAs and DHRGs in all AML patients.

**Supplementary Table 16.** Correlation coefficients between miRNAs and DHRGs in the high-risk group.

**Supplementary Table 17.** Correlation coefficients between miRNAs and DHRGs in the low-risk group.

**Supplementary Table 18.** Correlation coefficients between top 10 hallmark pathways and DHRGs in the high- and low-risk AML groups.
