# Supplementary figures and images for: Identification of hub genes and potential molecular mechanisms related to drug sensitivity in acute myeloid leukemia based on machine learning
Source: Front Pharmacol. 2024 Apr 8;15:1359832. doi: 10.3389/fphar.2024.1359832 (PMC11033397; doi:10.3389/fphar.2024.1359832)

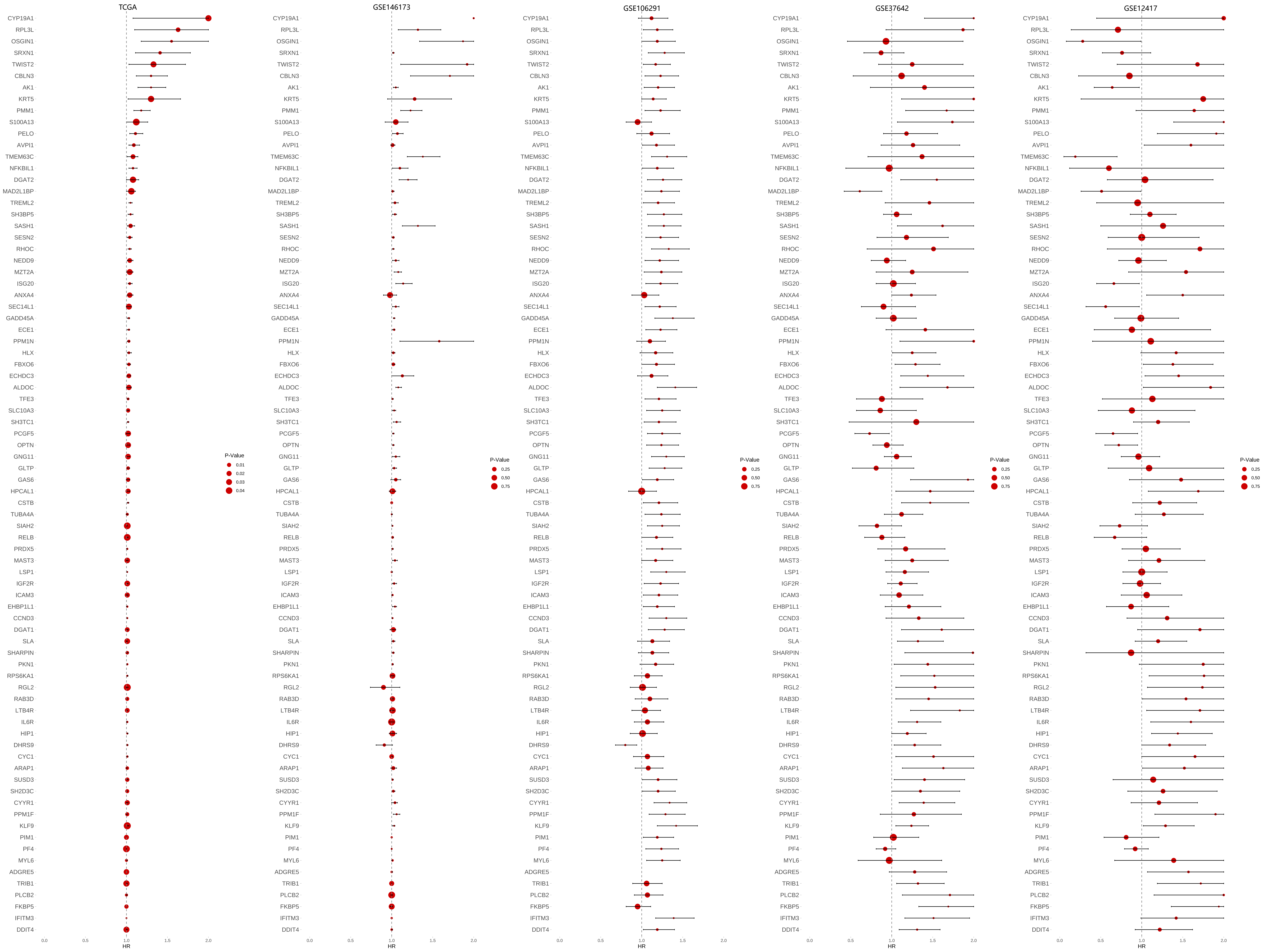

Supplement: Supplementary file 1 [file DataSheet1.zip › Additional files/SF1.pdf]

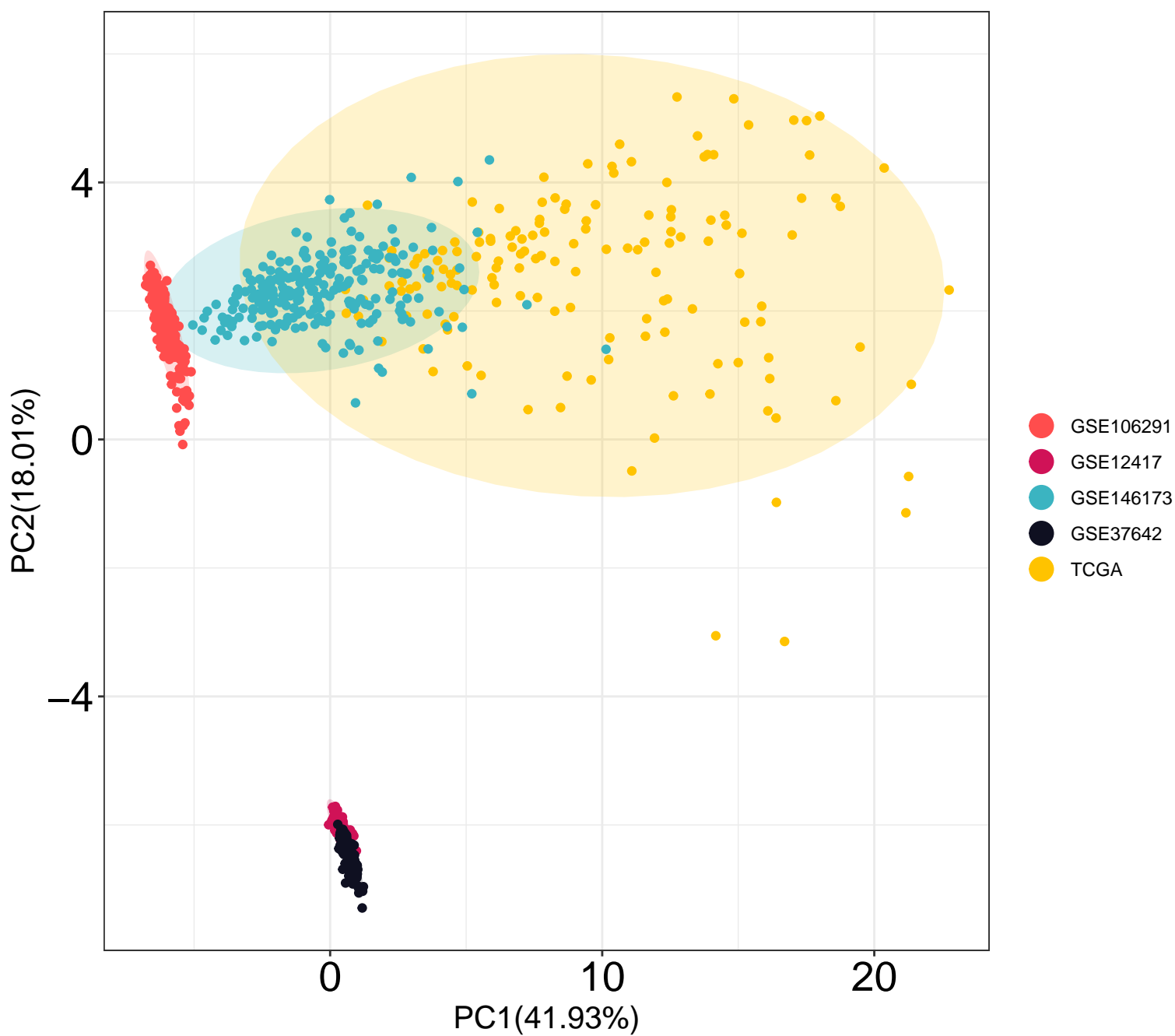

Supplement: Supplementary file 1 [file DataSheet1.zip › Additional files/SF2.pdf]

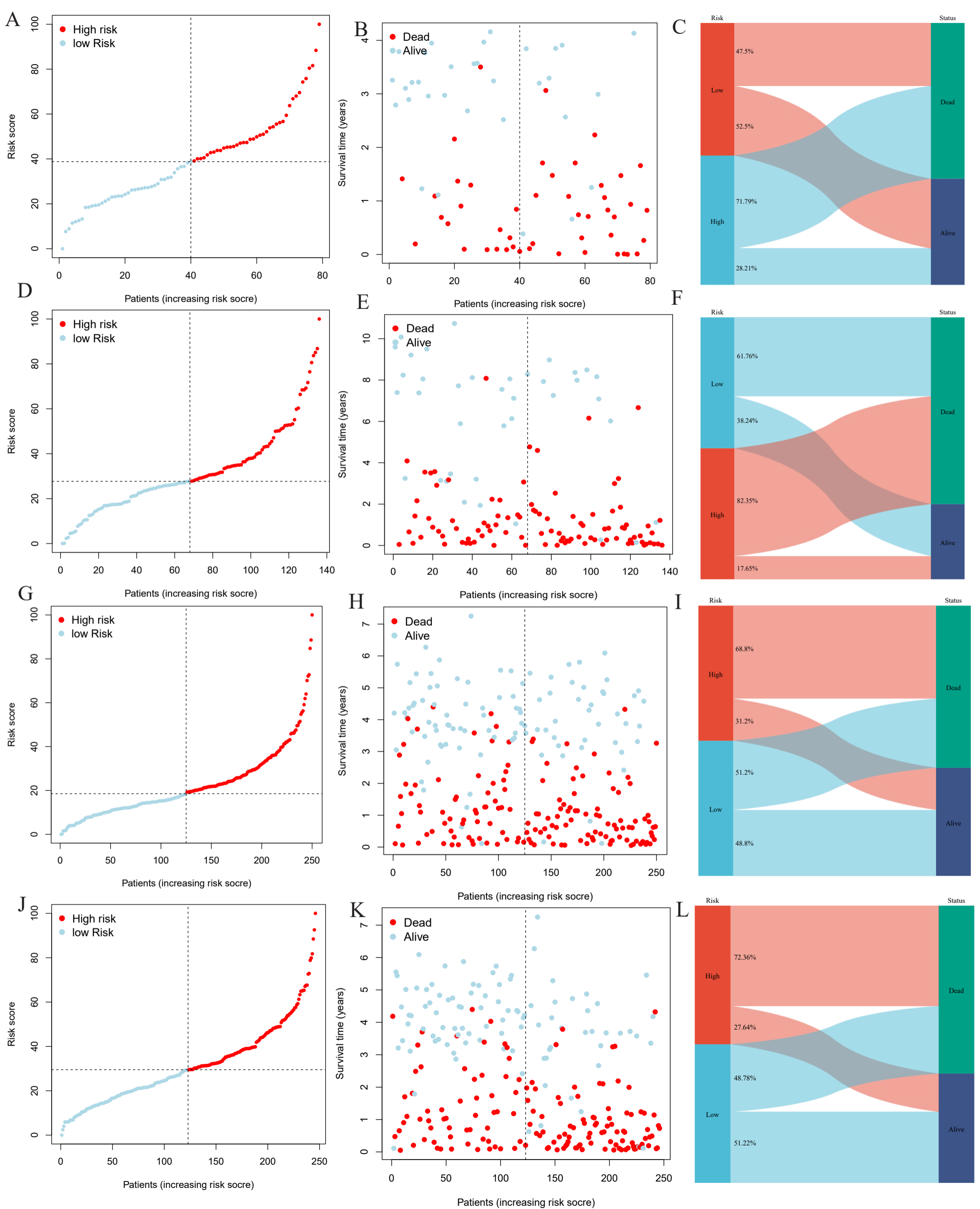

Supplement: Supplementary file 1 [file DataSheet1.zip › Additional files/SF3.pdf]

A

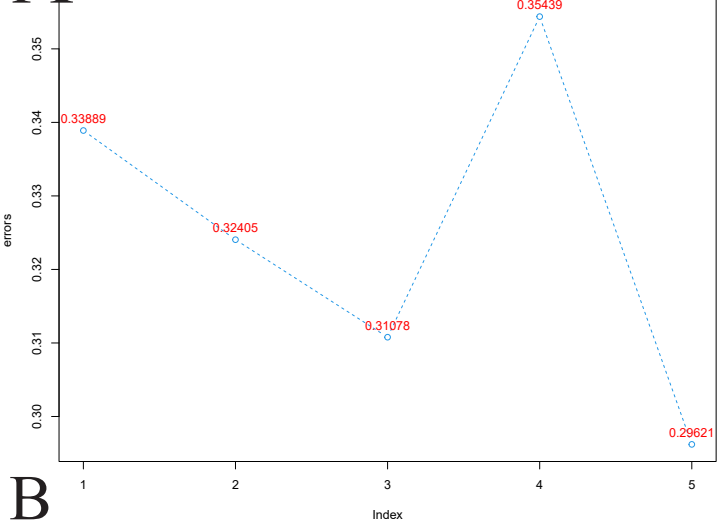

B

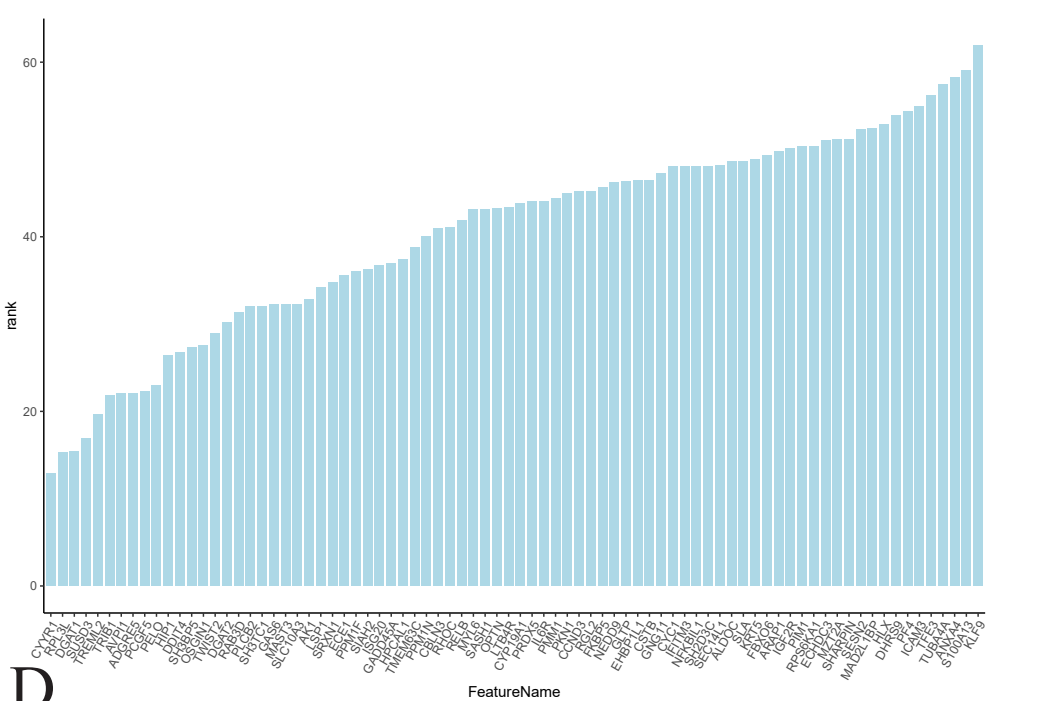

D

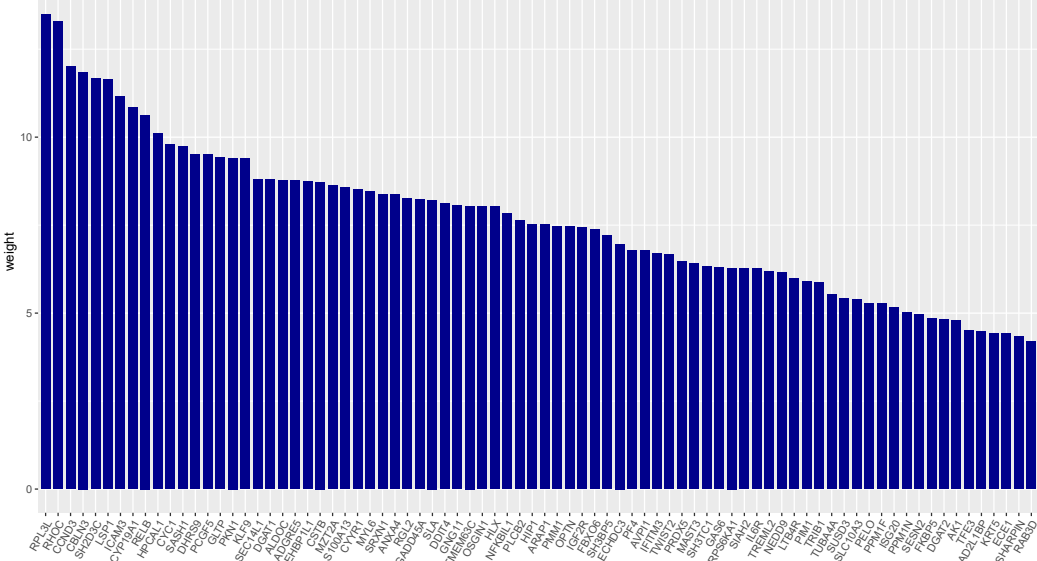

E

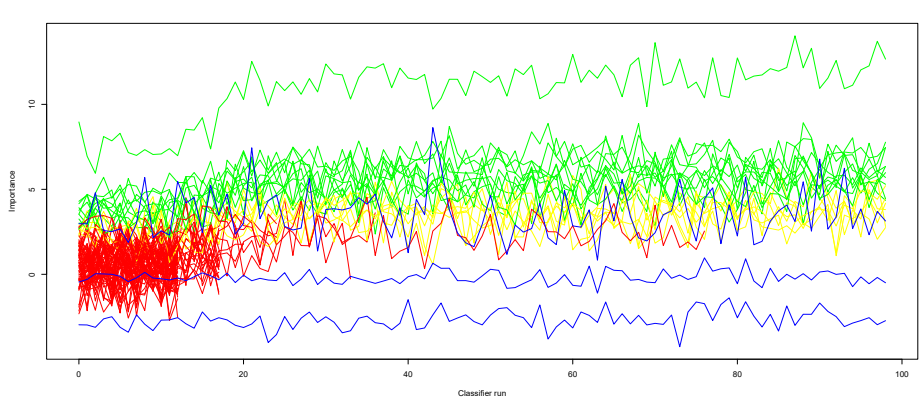

H

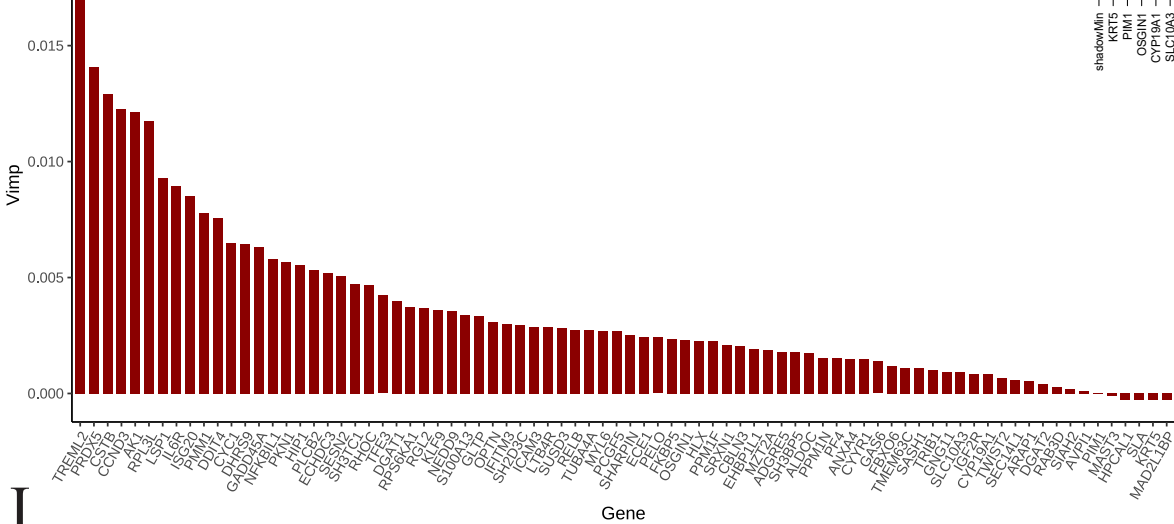

I

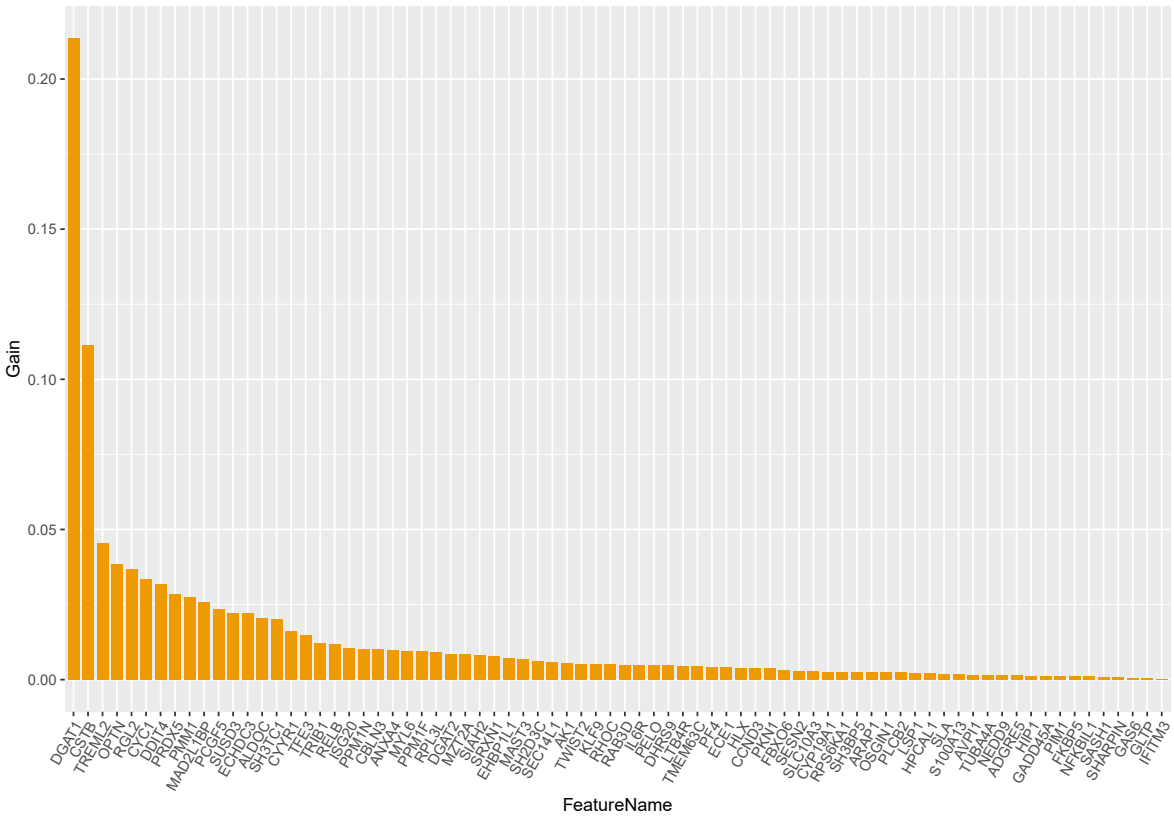

C

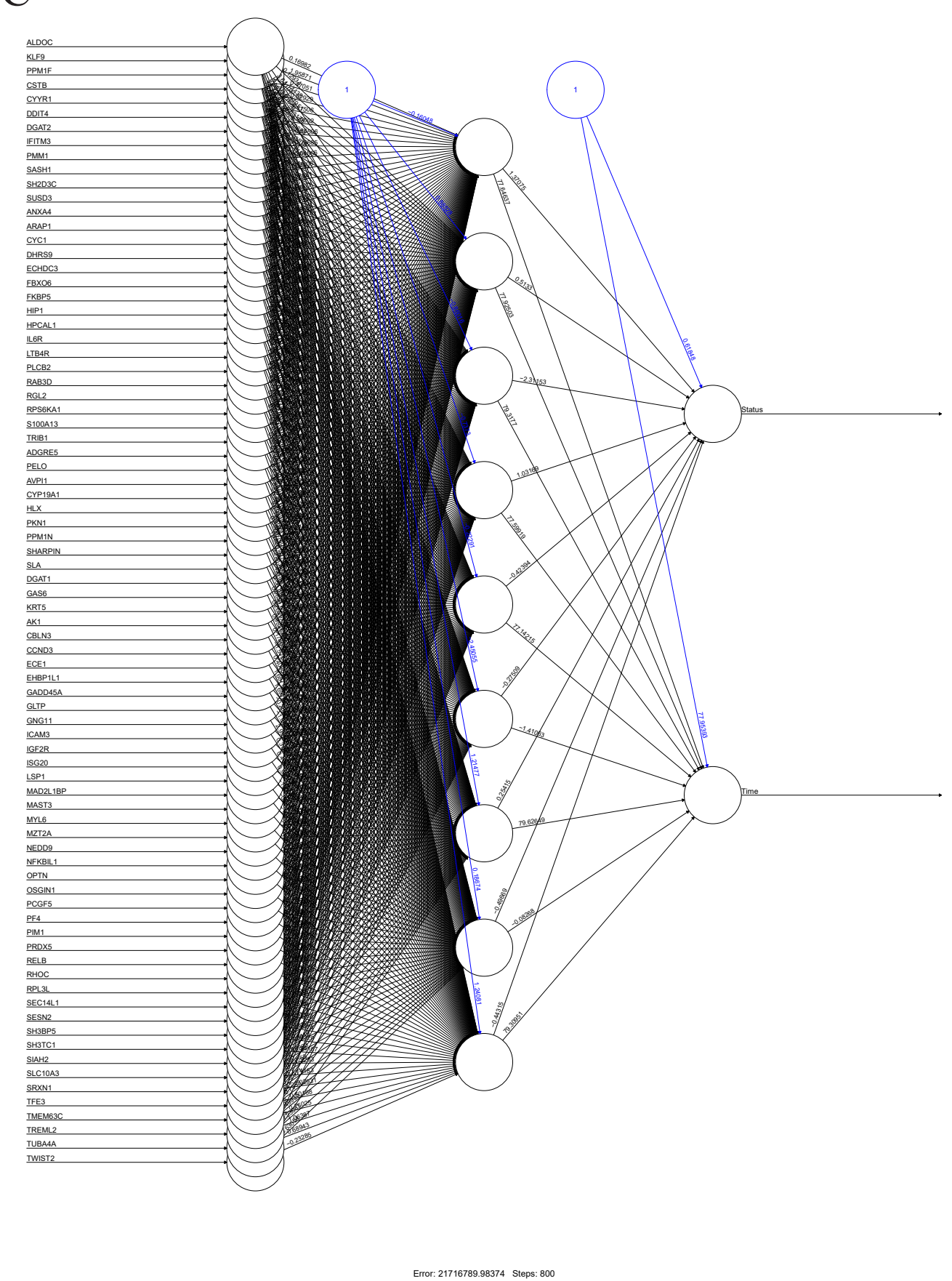

Error: 21716789.98374 Steps: 800

F

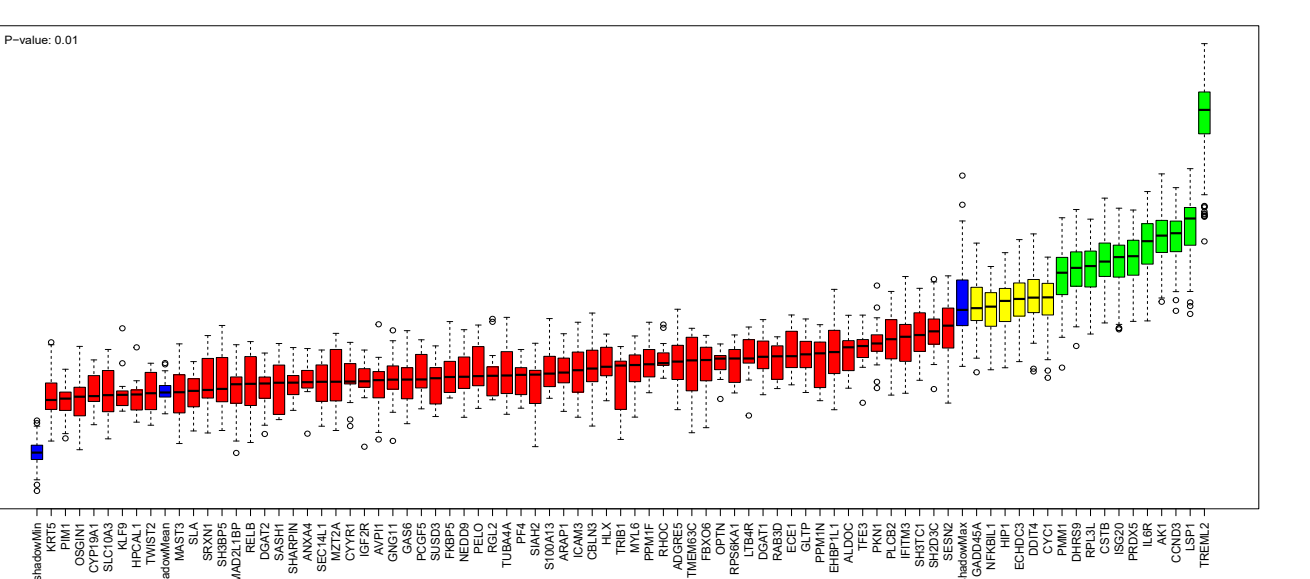

G

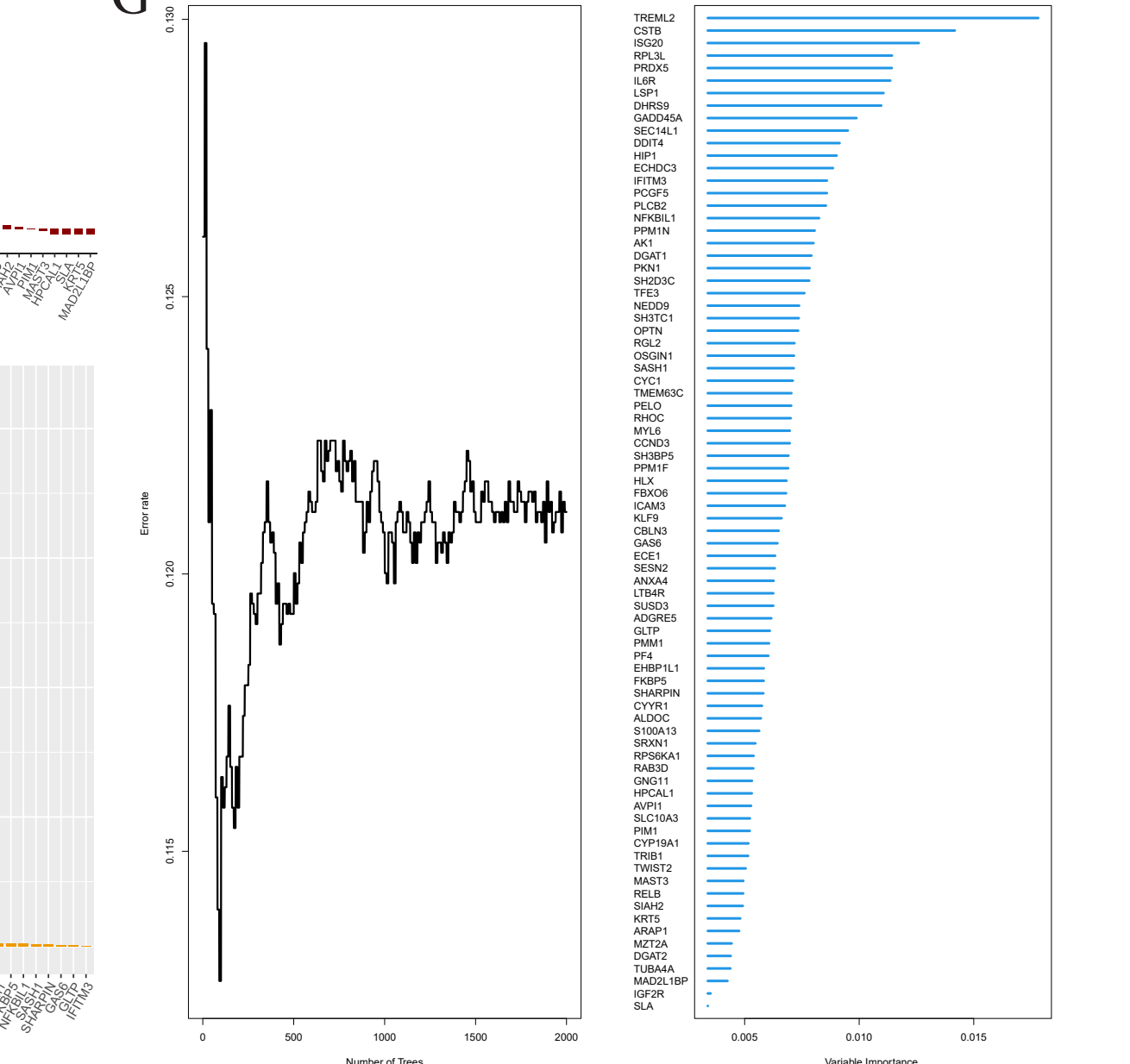

Supplement: Supplementary file 1 [file DataSheet1.zip › Additional files/SF4.pdf]

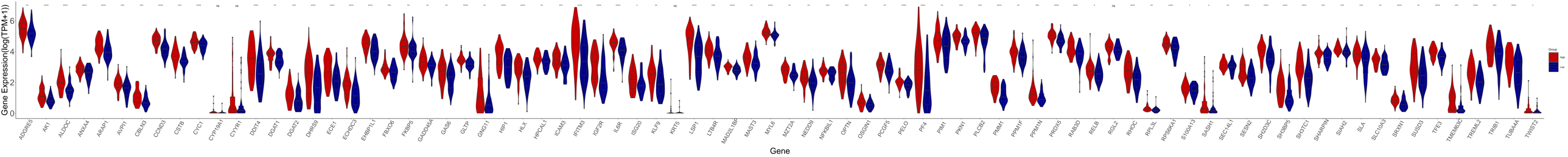

Supplement: Supplementary file 1 [file DataSheet1.zip › Additional files/SF5.pdf]

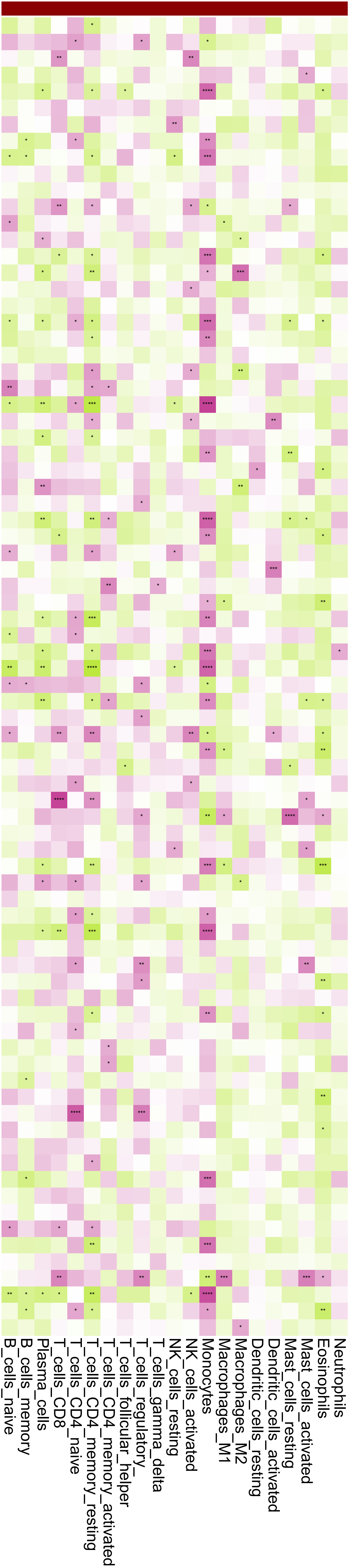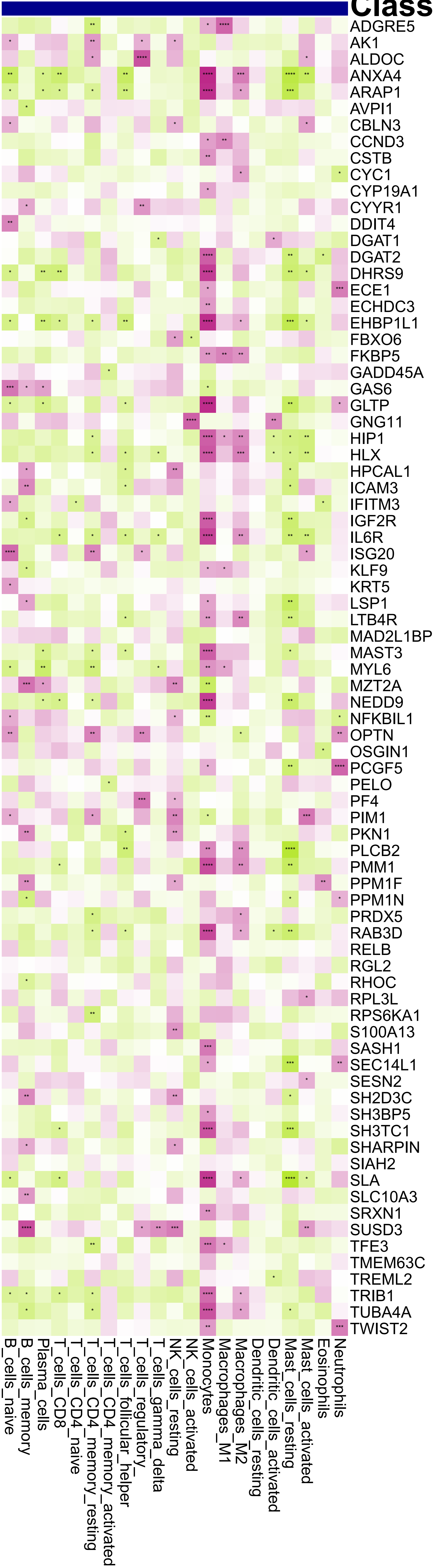

Supplement: Supplementary file 1 [file DataSheet1.zip › Additional files/SF6.pdf]
